# Sustainable Cold Mix Asphalt: A Comprehensive Review of Mechanical Innovations, Circular Economy Integration, Field Performance, and Decarbonization Pathways

**DOI:** 10.3390/ma18235452

**Published:** 2025-12-03

**Authors:** Muhammad Danyal Malik, Yongsheng Chen, Jian Mu, Ruikun Dong

**Affiliations:** 1 School of Civil Engineering, Chongqing University, Chongqing 400045, China; l2400460@stu.cqu.edu.cn; 2 State Key Laboratory of Safety and Resilience of Civil Engineering in Mountain Area, Chongqing University, Chongqing 400045, China; 3 Chongqing Communications Planning Survey & Design Institute Co., Ltd., Chongqing 400067, China

**Keywords:** cold mix asphalt, reclaimed asphalt pavement, sustainable pavement, emission reduction, performance enhancement

## Abstract

Climate change presents a major challenge of the Anthropocene, with construction activities contributing about 23% of global CO_2_ emissions. Pavement engineering, particularly hot mix asphalt (HMA) production, generates roughly 350 million tons of CO_2_ annually due to high-temperature processes. Cold mix asphalt (CMA) has emerged as a sustainable alternative, reducing energy use by 35–50% and emissions by 40–60% through ambient-temperature production with emulsified or cutback binders. Although early CMA formulations suffered from low mechanical strength, long curing times, and poor moisture resistance, recent innovations such as nano-modified binders, polymer and rubber additives, and optimized RAP utilization have greatly improved performance. Modern CMA now achieves enhanced rutting resistance (>4000 cycles/mm), moisture resistance (TSR > 85%), and rapid strength gain (24 h). This review synthesizes findings from over 160 studies to examine composition, property relationships, performance evaluation methods, life-cycle comparisons, and global field validations. Furthermore, it highlights gaps in predictive modeling, mix-design standardization, and circular economy integration to support the evolution of next-generation CMA technologies aligned with UN Sustainable Development Goals 9, 11, and 13.

## 1. Introduction

**Figure 1:**
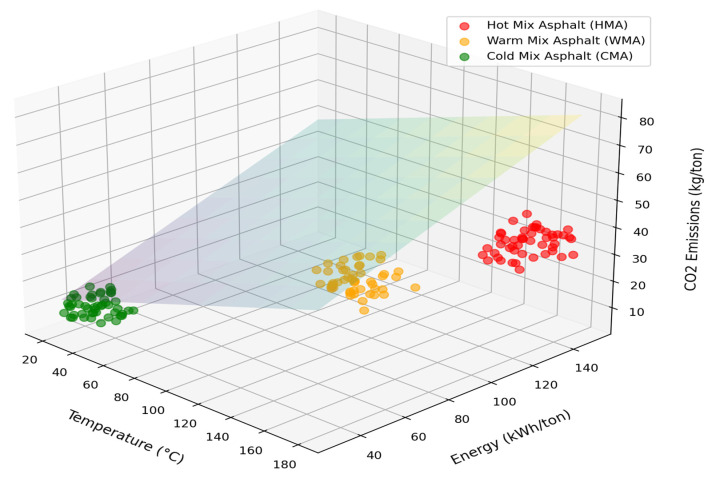
Categorization of asphalt mixtures by temperature [6].

The global road network, stretching over 64 million kilometers, is the backbone of nearly USD 28 trillion in annual economic activity [1,2]. However, the construction and maintenance of this infrastructure rely heavily on HMA, which accounts for approximately 95% of global asphalt production and raises significant environmental concerns. The production of HMA is an energy-intensive process, requiring mixing temperatures between 160 and 190 °C and emitting an estimated 53.6 kg of CO_2_ per ton of asphalt [3,4]. In response to the urgent need for decarbonization in the construction sector, CMA has emerged as a promising sustainable alternative. Produced at ambient temperatures (0–40 °C), CMA technologies can reduce energy consumption by 35–50% and carbon emissions by 40–60% compared to HMA [4]. Despite these clear environmental and economic advantages, the widespread adoption of CMA has been historically hindered by a significant contradiction: its reputation for poor mechanical performance [5]. As depicted in Figure 1, asphalt mixtures are typically classified by production temperature, and CMA stands out as a true zero-heat solution, produced at ambient temperature without requiring aggregate dryers or fuel-burning equipment [3,6,7]. Between these temperature extremes, warm mix asphalt (WMA) and half-warm mix asphalt (HWMA) represent intermediate technologies that offer partial environmental benefits. WMA, produced at temperatures between 100 and 140 °C, reduces mixing temperatures by 20–40 °C compared to HMA, resulting in meaningful energy savings and emission reductions [8]. Various technologies, including chemical additives and water-foaming techniques, enable this temperature reduction while maintaining acceptable performance. HWMA, produced below 100 °C, occupies the transitional zone between WMA and CMA, demonstrating good performance with lower environmental impact than conventional HMA [3]. Early CMA formulations were characterized by low Marshall stability (MS) values of 3.5–4.5 kN, which were 40–60% lower than typical HMA (8–12 kN), and a high susceptibility to moisture damage, with Tensile Strength Ratios (TSRs) often falling below the acceptable 70% threshold [5,6]. Furthermore, extended curing times of 28 to 90 days created logistical challenges, contrasting sharply with HMA, which can be ready for traffic within 24 h [9]. These limitations relegated CMA primarily to low-traffic roads and temporary patching applications; resistance has seen remarkable gains, with studies reporting TSR values consistently above 85% and, in some cases, even exceeding 100%, a phenomenon attributed to the synergistic effects of cement hydration and emulsion curing [8].

**Figure 2:**
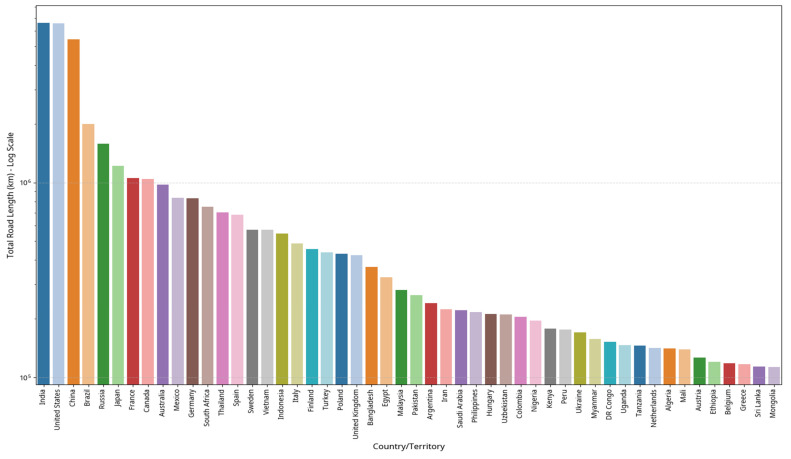
Road network length of the top 40 countries [13].

The urgency of transitioning to sustainable pavement technologies like CMA becomes evident when considering the scale of the global road infrastructure [5,10,11,12]. As shown in Figure 2, the road networks of major economies are vast, with China (5.4 million km), the United States (6.5 million km), and India (6.6 million km) collectively accounting for approximately 18 million kilometers of paved roads [13]. These networks contribute between 12 and 16% of national transportation sector greenhouse gas emissions. The potential impact of CMA adoption in these markets is substantial: the technology enables the incorporation of up to 100% reclaimed asphalt pavement (RAP), which could reduce virgin material demand by as much as 850 million tons annually while diverting approximately 320 million tons of pavement waste from landfills each year [12,13,14,15,16,17]. A notable example from the United States demonstrates this potential: in 2019, the asphalt industry utilized approximately 89.2 million tons of RAP, resulting in cost savings exceeding USD 3.3 billion, reductions of 2.4 million metric tons of CO_2_ (56–64% decrease), and energy savings of 40–60% compared to virgin materials [18]. A critical challenge in evaluating these advancements, however, lies in the methodological differences between studies. The field lacks a universally standardized mix design and curing protocol, making direct comparisons of performance data difficult. For example, some researchers employ accelerated curing at elevated temperatures (e.g., 60 °C for 2 days) to simulate long-term strength, while others use ambient curing for extended periods (e.g., 28 days at 23 °C) [7,11]. This discrepancy in methodology can lead to vastly different reported outcomes for similar mixtures and complicates the establishment of reliable performance benchmarks [8]. This review will comment on these differences and highlight the urgent need for a unified testing framework. Furthermore, the literature presents seemingly contradictory findings regarding the field performance of CMA. While some studies report early failures, such as the rapid deterioration of dense-graded CMA patches in severe winter conditions in China [10], others document exceptional long-term durability. For example, a CMA pavement with 70% RAP on Scotland’s A90 trunk road showed no significant distress after 10 years under heavy traffic (>10 million ESAs) [12], and another in Sweden remained in excellent condition after 15 years [14].

**Figure 3:**
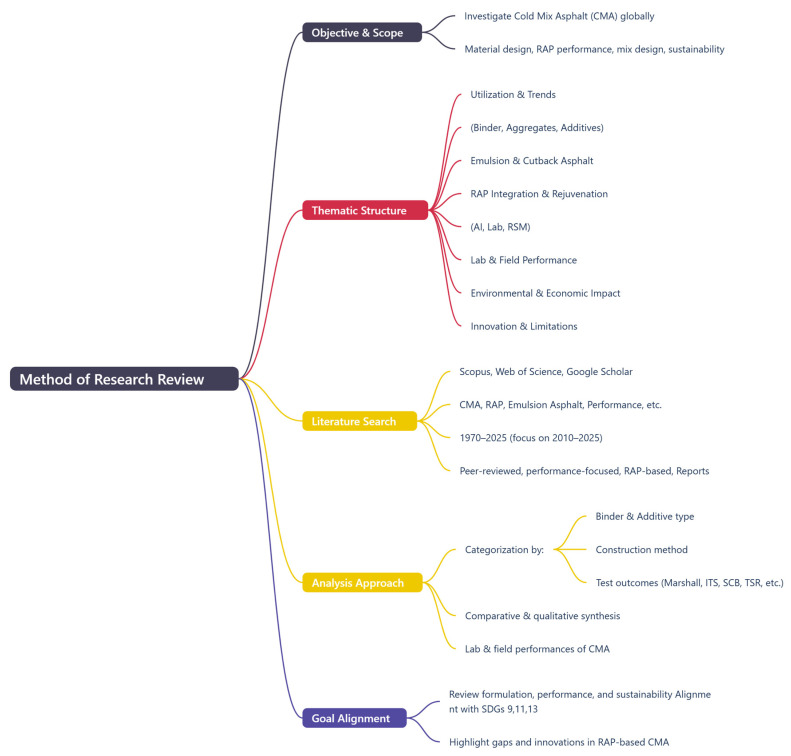
Method for research review.

This review will analyze such case studies to identify the underlying factors—such as mix design, climate-specific formulation, and construction quality—that determine success or failure. Figure 3 illustrates the framework adopted in this review for evaluating these recent technological improvements.

This paper aims to synthesize these disparate findings by providing the first multi-scale assessment of CMA systems through three interrelated perspectives:

- Material innovation: A chemo-rheological optimization of the binder-aggregate interface.
- Performance validation: An analysis of advanced characterization methods and a critical look at the methodological gaps in current testing standards.
- Comparative Sustainability metrics: A life-cycle assessment (LCA) of modern CMA compared to traditional HMA/WMA alternatives.

By comparing findings, identifying contradictions, and highlighting key trends, this research provides a critical and comprehensive overview to advance the scientific basis for CMA as a viable, mainstream, and sustainable road-building alternative, in alignment with the United Nations’ Sustainable Development Goals (SDGs) 9, 11, and 13.

## 2. Global Utilization and Economic–Environmental Rationale

Global CMA usage has increased by approximately 35% since 2015, driven by its environmental and economic advantages over HMA. CMA produces less greenhouse gas when produced and uses 56–64% less energy than HMA, which creates an opportunity for CMA to be used as a green and cost-effective paving material for the modern construction industry. The increase in use of CMA is most evident on low-volume roadways (less than 200 AADT), where it is currently being used as approximately 25% of all roadway pavement material in Europe and 15% of all roadway pavement material in North America [10,19].

In addition to providing several environmental advantages, CMA also provides many economic advantages to producers and users of CMA. Production costs are typically reported to be 20–30 percent lower than those of conventional HMA. The primary reasons for this price difference are due to the elimination of the need to heat the mixture prior to laying and reduced equipment-related capital costs [20]. A comprehensive study by Gu et al. [21] examined the differences in life-cycle costs of CCPR-F versus HMA and determined that CMA technologies can provide a savings of 32% in life-cycle costs over HMA (USD 16,224 vs. USD 23,971 per lane km), based upon reduced energy consumption, less use of equipment, and the ability to effectively incorporate RAP into the new product. In terms of environmental factors, CMA requires only 14–15 MJ/ton and produces 1–1.13 kg of CO_2_ per ton of product; HMA, by comparison, requires 275 MJ/ton and generates 22 kg of CO_2_ per ton of product. Additionally, a series of field studies conducted in Scandinavia (Sweden) [22] further show how CMA facilitates logistics, demonstrating that on-site CMA manufacturing can significantly decrease transportation-related emissions by 56–64% for projects greater than 50 km away from HMA plants. The above results directly align with European Union sustainability directives (EN 13108-31 [23]), thus enhancing CMA’s position as a green and sustainable method of producing pavements through resource-efficient methods.

Similarly, field testing on the Rv95 highway [22] demonstrates that CMA achieves stiffness levels equal to 101% of those of HMA (5965 MPa vs. 5906 MPa) and shows it is structurally durable over time (greater than 5 years) and under normal traffic conditions. Additionally, the local manufacture of CMA reduces logistics-related emissions, and the use of recycled asphalt (0–30%) assists in meeting circular economy goals. Most notably, Sweden’s cement-free CMA (4.2–4.8% bitumen emulsion) has been successful in cold climates, with no shrinkage problems and providing reliable, low-carbon pavement performance.

Although there are many benefits to using CMA, it is estimated that approximately 5–7% of the global asphalt output is in the form of CMA. The primary cause for this relatively low use of CMA is the mechanical reliability of earlier formulations of CMA. Earlier CMA formulations displayed low MS values (3.5–4.5 kN) compared with HMA’s 8–12 kN and showed poor moisture resistance (<70%), limiting their application mainly to low traffic roads (below 1000 AADT) [10,24]. However, recent advances in additive technology, such as incorporating 2–4% cementitious fillers or around 1.5% nano-silica, have significantly improved the mechanical stability and moisture resistance of CMA, helping to overcome these long-standing durability limitations [25,26]. For example, a study by M. Rezaei et al. [2,27] reported that using 100% RAP combined with a high-performance C60B5 cationic emulsion (slow-setting, containing rejuvenators), 2% Portland cement, and a dense-graded RE2 aggregate structure (in line with Spanish PG-4 standards [28]) produced a CMA-2 mixture with remarkable performance. This optimized mixture achieved a dry indirect tensile strength (ITS) of 1568 kPa and a wet ITS of 1662 kPa, yielding a TSR of 106%. The unusual strength increase upon moisture exposure was linked to the synergistic interaction between cement hydration, which continuously builds strength, and emulsion curing, which enhances binder adhesion. Moreover, the CMA-2 mixture attained an MS of 18.95 kN, approximately 65% higher than the reference value for conventional HMA (11.5 kN). The enhanced strength of CMA-2, along with its high resistance to degradation caused by water, makes it suitable for very heavy traffic areas and those that are prone to excessive moisture. As such, this improvement will redefine what is expected from cold-recycled materials in terms of performance capabilities, thereby allowing for the reliable placement of cold-recycled materials on highways carrying between 5000 and 10,000 AADT [27,29,30,31].

**Figure 4:**
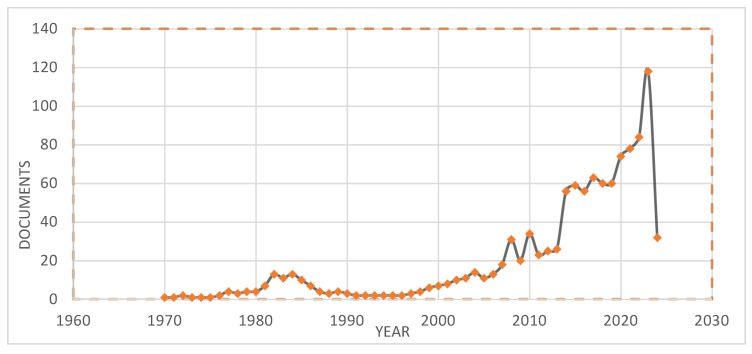
Annual publication trend of CMA research [19].

**Figure 5:**
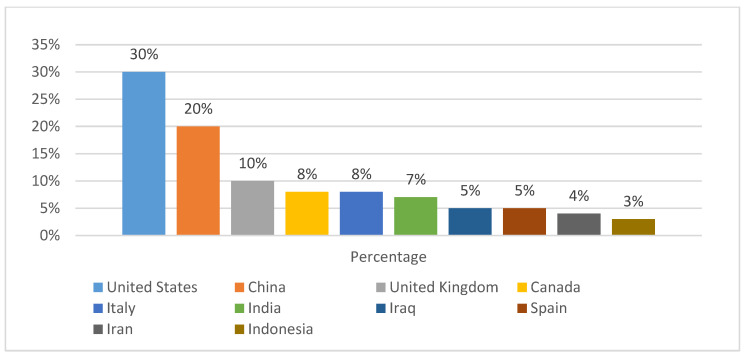
Number of research documents on CMA by country [19].

As for research development, bibliometric analysis indicates an elevated level of academic interest and involvement in CMA technologies. Approximately 57% of the 980 CMA-related papers indexed in Scopus (1970–2024) were published after 2010, representing increasing worldwide interest in CMA technology, as illustrated in Figure 4. Similarly, Figure 5 indicates that there are several differences in regional research outputs for CMA. The top countries for CMA research include the U.S., China, and India, with 30%, 20%, and 7%, respectively. The U.S., China, and India have national plans to reduce carbon emissions from transportation systems (the U.S. has 6.5 million km of highways; China has 5.4 million km of highways; and India has 6.6 million km of highways). The regional distribution clearly shows regional innovation clusters emerging worldwide. Each cluster will contribute its own unique approach to advance CMA technology and improve the environmental performance of highways. European research focuses on using 100% RAP, with a goal of creating circular economies within the industry. Conversely, North America has focused on developing new rapid-curing bio-rejuvenators to enhance the mechanical properties of hot mix asphalt during the initial stages of construction and accelerate construction timelines in areas with cold or wet climates [10,19]. The differing trends across regions are an example of how global collaboration and specialization are leading to a next generation of sustainable pavement innovations.

## 3. Material Composition and Chemo-Rheological Design

Performance of CMA is based upon how well the three major components, the binder, the additive(s), and the aggregate(s), interact. All three components contribute in different ways to the chemo-rheological properties, the durability, and the structural integrity of the final mixture:

1. Aggregates: Aggregates form the backbone of CMA and are carefully selected to achieve an optimal gradation, typically with a nominal maximum size below 12.5 mm. The particle size distribution generally follows established gradation frameworks, such as LB-10 or UPM-13, ensuring a dense, stable aggregate matrix. Incorporating 4–8% limestone aggregate (≤0.075 mm) into the mix will result in an increase in packing density to greater than 78%, which can result in better load transfer and improved rigidity. Angular crushed aggregate with a Los Angeles (LA) abrasion value less than 25% provides superior interlock characteristics and superior rut-resistant characteristics under traffic conditions [32].
2. Binder: Binder is the binding agent that holds the aggregate together; binder is usually an emulsified asphalt (cationic CSS-1h) or cutback asphalt (MC-70) with a bitumen content between 60 and 70%. The binders are engineered to exhibit predictable failure behavior, enabling demulsification within 30 min at 25 °C for proper application and early strength development. Bio-based diluents are often incorporated to improve workability at ambient temperature by decreasing viscosity from 10–20 cP to 1–3 Pa·s at 25 °C, while providing a minimum flash point of 200 °C to ensure a safe, performant product [4,33].
3. Additives: A range of polymeric and nano-scale modifiers are employed to strengthen the cohesion–adhesion balance of the mix [34]. Common examples include SBS elastomers (around 2% by weight) and nano-clay (0.5–1.5% by weight), both of which can boost Marshall stability from 0.5–1.5 kN in unmodified CMA to 4–12 kN in modified mixtures. Anti-stripping agents, such as hydrated lime (1–2% by weight), are also incorporated to improve moisture resistance, helping the mix retain a TSR of over 85% after 24 h of water immersion [35,36].

### 3.1. Emulsion Chemistry and Its Influence on CMA Performance

The performance of CMA is fundamentally governed by the properties of the asphalt emulsion used as a binder. An asphalt emulsion is a stable dispersion of fine bitumen droplets (typically 0.1–10 μm in diameter) within a water-based medium, stabilized by a small amount of a surfactant (0.1–2.5%). While the chemistry can be complex, its practical implications for CMA performance can be understood by focusing on three key characteristics: the breaking mechanism, the electrical charge, and the formulation [32,37,38,39].

#### 3.1.1. Emulsion Type and Electrical Charge

**Figure 6:**
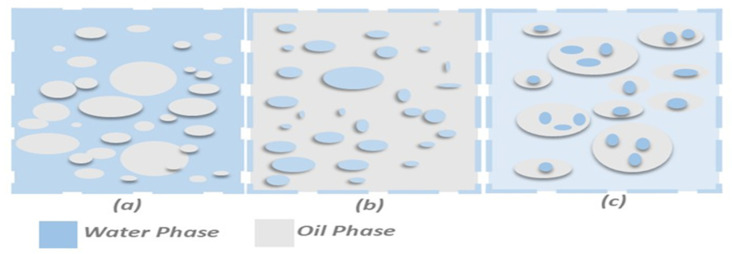
Types of emulsion: (**a**) oil in water, (**b**) water in oil, and (**c**) multiple.

Asphalt emulsions are generally categorized into oil-in-water (O/W), water-in-oil (W/O), and multiple (W/O/W) systems, as illustrated in Figure 6. For CMA, O/W emulsions are standard because the continuous water phase provides enhanced stability and allows proper coating and workability during mixing. Surfactants impart an electrical charge to bitumen droplets, producing either of the following:

- Cationic emulsions: ζ ≈ +40 to +70 mV, highly compatible with most aggregates (siliceous, granitic) due to their natural negative surface charge [20,40].
- Anionic emulsions: ζ ≈ −40 to −60 mV, best suited for positively charged calcareous aggregates such as limestone [20,40].

Correct charge–aggregate matching is essential for moisture resistance and prevention of stripping, making cationic slow-setting (CSS/SS) emulsions the dominant choice for CMA [32,34,35,36,37,38,39].

#### 3.1.2. Breaking Mechanism and Curing Behavior

The most critical performance-related property of an emulsion is its breaking speed, which refers to the process by which the bitumen droplets coalesce and separate from the water phase to form a continuous binder film [41,42]. This process directly controls the curing time and early strength development of the CMA. The breaking process occurs in stages, beginning with flocculation (droplets clustering together) and ending with curing (droplets merging into a film) as water evaporates [38,39,41].

**Figure 7:**
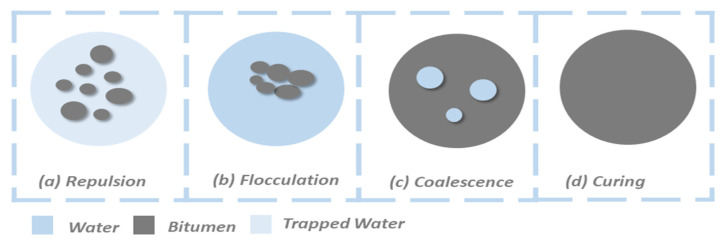
Stages of emulsion separation.

Breaking in bitumen emulsions occurs through three overlapping stages, mainly controlled by surface charge (ζ-potential), as illustrated in Figure 7:

- Stage I-Flocculation: As water dilutes the surfactant, the zeta potential drops from stable ranges (+40–70 mV for cationic and −40–60 mV for anionic) to a critical level (~20–25 mV). Reduced electrostatic repulsion allows droplets to collide under Brownian motion, requiring <10 kT activation energy [41,42,43].
- Stage II-Coalescence: When the flocculated droplets come into contact with each other, a thin layer of water forms between them. The film will collapse when the capillary pressure is greater than 5 kPa. At that point, the residual zeta potential (approximately ±10–20 mV) will not be enough to prevent the droplets from merging more rapidly [41,42,43].
- Stage III-Curing: In the final stage of this process, the remaining water between the droplets will evaporate, and the binder will redistribute until the asphalt film has solidified. If there is too much humidity (greater than 90%), then the curing of the asphalt film may take two to three times as long because the retained moisture will delay the complete coalescence of the droplets [41,42,43].

The speed of this process is engineered by selecting a specific type and concentration of surfactant, which classifies emulsions as rapid-setting (RS), medium-setting (MS), or slow-setting (SS). For CMA applications, slow-setting (SS) emulsions are almost always used. This is because a slow break is essential to ensure there is sufficient time to mix the emulsion with the aggregate, transport the mix to the site, and compact it before the binder becomes too stiff. A premature break would result in a poor-quality, unworkable mix [37,38,39,41,42,43].

#### 3.1.3. Formulation and Performance-Enhancing Additives

Beyond the basic chemistry, modern emulsions are complex, engineered systems that often include a range of additives to enhance the performance of the final CMA. These include:

- Polymers: Styrene-Butadiene-Styrene (SBS) and other polymers are frequently incorporated into the base bitumen before emulsification. When the polymer is mixed into the bitumen, the polymer creates a network in the binder, which increases the binder’s elastic properties, increases rutting resistance, and extends the binder’s fatigue life [29,41].
- Adhesion Promoters: After choosing the right charge for the emulsion, chemicals called anti-stripping agents are commonly used to increase the adhesion between the aggregate and binder and to improve water resistance [41].
- Fillers and Modifiers: Active filler materials such as cement and hydrated lime are often added to the CMA mixture. The active fillers have to be considered when creating the emulsion to ensure that there is no premature breakdown of the emulsion due to interaction with the additive [44,45,46].

### 3.2. Global Emulsion Standards and Innovations

Bitumen emulsions are characterized by diverse formulations developed to meet specific application needs and environmental conditions. Regionally formulated bitumen emulsions are formulated to best match local aggregate and climates but contain 60–65% residual bitumen and cure at ambient temperature. Regional examples highlight the variations in these formulations.

- Nordic (Sweden):

CMAs containing 2% BA-modified emulsions (with B160/220 and B330/430 binders having a softening point of 33–39 °C) have exhibited excellent durability on Sweden’s rural roads for 15+ years. CMA exhibits very low temperatures (−35 °C), and despite having a high void content, it consumes less than half of the diesel consumed by conventional HRA (0.12 L diesel/ton). Additionally, this formulation demonstrates less aging of the binder [29].

- Western Europe:

France: A Polyamine Emulsifier (PAE)-modified bitumen emulsion (BE) (penetration 160/220, 38.8 °C softening point, +35 mV zeta potential) has been developed [30].

Germany: Cationic emulsifier (CE)-series emulsions (e.g., CE-20/30: 60 °C softening point, +55 mV zeta potential) utilize emulsifier-driven thermal tuning, resulting in a 22% reduction in activation energy (with capillary pressure > 1.2 kPa) [38].

UK: Cement-modified Nymuls CP 50 (containing 3% cement, achieving 1850 MPa stiffness with 30% RAP) and fast-setting anionic emulsions (64 °C softening point) are employed to facilitate rapid urban repairs [47].

USA: A CSS-1h emulsion (58.2% residual asphalt) has enabled the production of dense-graded CMA with 12% air voids (compared to 7% design content) and workable compaction using perforated molds. Variants such as CSS-1hl and CSS-1h + 3% cement demonstrated enhanced moisture resistance, with a TSR up to 0.99 [48].

- Southeast Asia:

Malaysia: ACP-DMT emulsion, modified with 2% cement and 1% nano-silica, exhibits resistance to over 5000 cycles/mm in permanent deformation testing (PDT) (61.8 °C softening point) [49].

China: ACP-DMT emulsion, modified with 2% cement and 1% nano-silica, exhibits resistance to over 5000 cycles/mm in PDT (61.8 °C softening point) [33].

Emerging technologies are also contributing to emulsion innovation.

- Graphene

The incorporation of graphene oxide (GO) into cold mix epoxy asphalt enhances compatibility and thermal stability (0.1 wt.% GO is optimal for heat resistance). It also improves toughness while lowering the glass transition temperature, thereby increasing flexibility. Quantitatively, the Elongation at Break, a key indicator of flexibility and fatigue resistance, increases significantly from approximately 65% in the control sample to about 90% with 0.2 wt.% GO, representing a nearly 38% improvement. However, trade-offs include reduced strength and storage modulus, with the tensile strength decreasing slightly from approximately 2.4 MPa in the control to 1.9 MPa at 0.2 wt.% GO. Despite the slight reduction in strength, the substantial gain in flexibility suggests an extended service life [50].

- Microencapsulated bio-rejuvenators:

pH-triggered (>9.5) microencapsulated bio-rejuvenators are being synchronized with asphalt emulsions (typically pH 10–12) to facilitate staged binder reactivation in CMA applications [50].

Unmodified cationic slow-breaking emulsions from Sweden (CSB-N630/CSB-N240, modified with 2% BA on Pen 160/220 and 330/430 binders, yield softening points of 33 °C and 39 °C, respectively. In France, a PAE-emulsified BE with a Pen 160/220 binder achieves a 38.8 °C softening point. German CE-series emulsions (Pen 20/30, 50/70, and 70/100), stabilized by cationic emulsifiers, exhibit graded softening points ranging from 48.1 °C up to 60 °C. This demonstrates how binder penetration grade and emulsifier selection alone can tailor thermal performance without requiring polymer modification [20]. The UK Nynas emulsion is specified with a penetration grade of 40/60 and a softening point range of 48–56 °C. In contrast, mixed emulsions incorporate various additives to enhance performance [51]. Global examples, including the UK’s Nymuls CP 50, Sweden’s modified anionic blends, the USA’s cationic slow-setting variants, Malaysia’s ACP-DMT, and China’s formulations, integrate additives such as cement, rosin, and limestone, often at approximately 60% bitumen content, to improve durability, strength, and adaptability for construction purposes [47,51].

### 3.3. Cutback Asphalt: Composition, Classification, and Applications

Cutback asphalt is made by using an asphalt cement and a solvent to create a lower viscosity product than regular asphalt cement, making it easier to apply in low temperature conditions. Additionally, this process helps cutback asphalt be applied with aggregate materials, as it does not need to be heated, providing many benefits for pavement construction in cold weather conditions [42,51,52].

Cutback asphalt generally includes three components: asphalt cement and a solvent (most commonly derived from petroleum) and may include other types of additives to provide improved performance characteristics. Due to these properties, cutback asphalt can be used for a variety of different paving projects and is especially useful in colder climates. Cutback asphalt is categorized into three classes based upon the solvent evaporation rate: rapid curing (RC), medium Curing (MC), and slow curing (SC). MC cutbacks are formulated with a kerosene-based solvent (ASTM D 2027 [53], AASHTO M 82 [54]), and SC cutbacks are created through distillation of crude oil or through a process called “fluxing” of asphalt with a lighter oil (ASTM D 2026 [55]). Numerical suffixes indicate viscosity; for example, MC-250 represents a minimum viscosity of 250 centistokes at 60 degrees Celsius [20,56,57].

The curing rate largely determines the intended application:

- RC: Contains volatile solvents that evaporate quickly, allowing traffic to return rapidly. Ideal for patching and surface treatments.
- MC: Features a moderate evaporation rate, providing good workability and effectiveness for base courses and surface treatments [58].
- SC: Contains minimal volatile solvents, giving extended work time and suitability for prime coat applications and CMA treatments.

Solvent-specific performance and application of cutback asphalt are directly dependent upon the amount of that solvent used for the development of a particular cutback asphalt formulation. Solvents can be adjusted to improve asphalt workability and to alter the behavior during application. Due to high adhesive quality with aggregate materials, the use of cutback asphalt produces a durable and flexible asphalt mixture. An automated application method for cutback asphalt (without heat) provides an excellent solution for cold-weather maintenance and emergency repairs, enabling rapid execution of these activities [12,20,24].

Cutback asphalt is utilized extensively in the construction of pavements for the repair of potholes and surface treatments and as a component in CMA. These applications are made possible by the rapid curing and application of cutback asphalt, which can be used at low temperatures. Its low temperature use makes it ideal for various maintenance operations. However, the use of cutback asphalts consumes high amounts of oxygen, causing the generation of VOC that can lead to air pollution and human health hazards [57]. Disadvantages of cutback asphalt also include the risk of fire, high fuel consumption during combustion, and poor adhesion to wet surfaces. However, in spite of these disadvantages, cutback asphalt can be of much value in emergency repairs and repairs during very cold weather [56,57,58].

### 3.4. Aggregate and Water Composition

The nature of the aggregates and the water content in CMA have a serious influence on its moisture resistance, durability, and environmental impact. The use of good aggregates with water management methods can minimize moisture damage to a great extent, thus prolonging the life and enhancing the durability and sustainability of the materials [44,59]. The addition of recycled material, RAP, and other waste products to the CMA improves its durability, allowing a safe performance under varying traffic loads [44,59,60]. In addition, the extended life of pavements reduces waste and emissions and allows for a construction method for roads that is environmentally sound. The use of sustainable resources and lower processing temperatures in the manufacture of CMA leads to lower greenhouse gas emissions, thus placing CMA as an eco-friendly means of constructing roads [39,44,59].

Water, in CMA, is a key constituent, since the lubrication of the aggregates is aided, allowing improved workability and better coating of the aggregate particles. The characteristics of the water used in the manufacture of CMA particles are also of a critical nature because of their performance and stability effects on the bitumen emulsions. The water should be clear of contaminants such as iron oxide, silt, magnesium, and calcium carbonate. Water that has calcium contents of less than 75 ppm is regarded as soft water, which is of a beneficial nature in CMA manufacturing, since the use of clean or potable water gives superior results. In conclusion, both the choice of aggregates used and the water content are vital to the improvement of the performance and sustainability of CMA in any infrastructure project [39,59].

CMA uses a wide variety of aggregates, such as the following:

- RAP: The use of recycled materials as ingredients in CMA is important for sustainability. Evidence suggests that RAP will also significantly improve the mechanical properties of CMA, such as stability and moisture resistance [39,61].
- Natural Aggregates: Traditionally, aggregates such as limestone and granite are mostly used. The choice of natural aggregate is important for the overall performance of the asphalt mix, especially in durability and moisture resistance [60,61].
- Waste Materials: There are several recent studies directed to the use of waste materials such as fly ash (FA) and waste cooking oil (WEO), as partial replacements for conventional aggregates. They also help with sustainability and can improve the performance properties of CMA [62,63,64].

## 4. Production and Storage of CMA

CMA is a type of asphalt designed for temporary repairs and low-traffic areas. It is produced by mixing aggregates, such as crushed stone and gravel, with a liquid bitumen binder, which can be in the form of a bitumen emulsion or cutback bitumen. This mixture is flexible for use and suitable for in-place or mobile mixing, enabling immediate use. Generally, because mixing temperatures are so low (10–25 °C) compared to HMA (150–170 °C) [65,66,67,68], the CMA can be made at a temperature of 10° C to 40 °C to reduce the amount of energy that is required and also make it possible to use higher percentages of reclaimed content; therefore, the majority of cold mix is used in low traffic applications such as pothole repairs because the reduced service life of the cold mix is acceptable in these types of applications [65,69]. To slow down the premature curing process, additives are added to the cold mix (cement is added at 1–3% and lime is added at 2%), and the cold mix is kept at an insulated temperature during storage/transportation [65,69]. The production of cold mix is completed through either the mixed-in-place method or the central-plant method [67,68,69].

### 4.1. Mixed-in-Place (MIP) CMA

**Figure 8:**
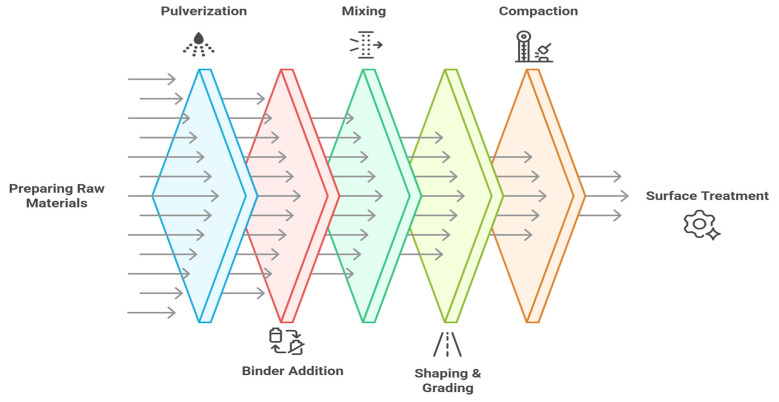
Mixed-in-place recycling.

The MIP CMA is a sustainable on-site pavement rehabilitation method using modern equipment and detailed materials management. The technique consists of aggregate preparation. The aggregates may be virgin materials or RAP and are stored in covered storage containers to avoid moisture absorption. The liquid binder is either emulsified or cutback asphalt and is stored in insulated tanks and applied by means of integrated spray bars on either rotary mixers or external asphalt distributors [70]. Rotary mixers consist of high-speed rotating shafts and blades to mix binder and aggregates in a chamber approximately 2 m in width and variable depth up to 250 mm. The mixture is applied uniformly through tailboard and hood adjustments. Milling and planning machines incorporating spray systems may similarly inject RAP or virgin aggregates so that the mixture is discharged either directly onto pavers or into windrows. Pre-wetting the aggregates with a detached water distributor incorporates additional moisture into the aggregates to increase the adhesion of the binder. The binder is applied to aggregates in quantities calculated to produce the correct rates [71]. In order to gain maximum benefits from the above, it is necessary to ensure that the equipment utilized is correctly calibrated, thus ensuring the amount of mixing is uniform. Monitoring the moisture in all respects, together with temperatures at which the materials are subjected to traffic during application, is also necessary. This method is beneficial not only in reducing emissions and the dependence on virgin materials but also in extending the life of pavements. It is an environmental option for on-site repairs such as potholes and utility cuts [65]. Figure 8 demonstrates the process steps for mixed-in-place CMA. The process for mixed-in-place CMA starts with a pulverization stage, in which the asphalt layer is granulated by means of a milling machine to produce a uniform base layer. Following this, the addition of binder as well as water commences, whereby bitumen emulsion or bitumen foam and water are injected into the mixing chamber to improve the binding characteristics of the mixture. Mixing then occurs, whereby a milling and mixing rotor rotates the granulated material to mix it uniformly with the binder and water to produce a consistent mixture. The mixed material is then shaped and graded in preparation for compaction with rollers to achieve the required density. The surface treatment to be applied is either chip seal or slurry seal, which will improve ride quality and durability on the surface. Figure 8 displays the process steps for mixed-in-place CMA [65,71].

### 4.2. Central-Plant Mixing (CPM) CMA

**Figure 9:**
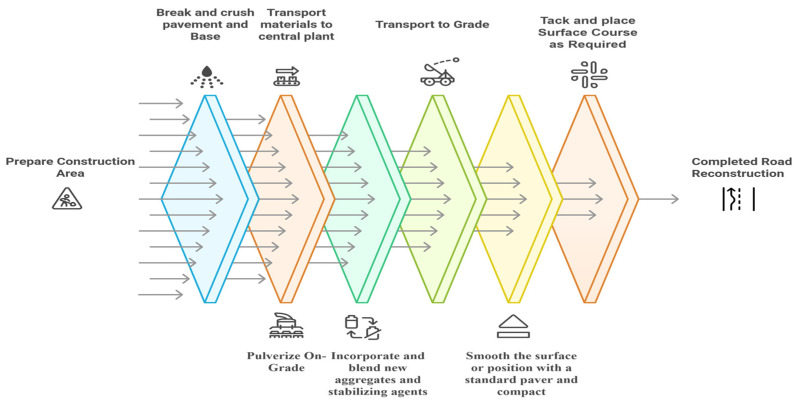
Central-plant recycling.

Centralized production of CMA is an effective and sustainable method that integrates RAP with new asphalt binders and recycling agents to create cold-base mixtures. This approach is particularly suitable for projects requiring high production rates and precise mix control. The system eliminates the need for heating; therefore, it reduces energy usage and greenhouse gas emissions associated with the typical method of production [71]. The steps involved in the central-plant mixing process for recycling are shown in Figure 9. The first step includes removal of existing pavement materials using a variety of methods that include milling, ripping, or scarifying [72]. Once the materials have been removed, they are hauled to the Central Facility for crushing and screening and then placed into storage for use on current or future projects. The importance of stockpiling is critical in preventing moisture from entering the pavement materials, thus affecting the overall quality of the material. Continuous mixing is an efficient means of producing asphalt because the continuous process has no interruptions.

The continuous process allows for the combination of RAP with virgin asphalt binders and additives in proportionate amounts in order to obtain the proper blending of the materials [66]. Quality assurance of performance is achieved through production quality control processes, including analysis of gradations of the material and methods of verifying the binder content [67]. Once the CMA has been produced, it will be hauled to the construction site, where it will be applied by standard paving equipment. Prior to compacting the CMA, it may be necessary to aerate the CMA using static steel wheel and pneumatic tire rollers to obtain the proper density and stability of the material [70].

The primary advantage of the central-plant CMA production system is its ability to provide improved quality control, increased efficiency in the implementation of the CMA, and environmental benefits from reduced energy usage and emission output. The central-plant CMA production system is effective in repairing pavement distress, such as reflection cracking and surface irregularities, while maintaining the geometric configuration of the pavement [70,71].

### 4.3. Construction Process of CMA

**Figure 10:**
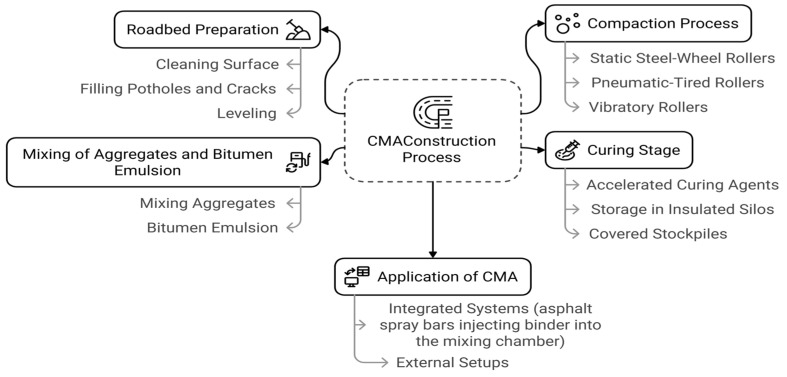
CMA construction process.

CMA construction starts with clearing and preparing the base, including removal of debris and leveling, as well as crack sealing, as shown in Figure 10. This will provide a solid base on which to lay the cold asphalt material. The next step is to apply the ambient temperature material manually or using a paver at temperatures of 10–30 °C, thus avoiding the need for heat-producing machinery [65,67].

CMA is made from emulsion-based materials (both anionic and cationic) and can be mixed and applied between 10 and 70 °C and produce less than 50 g VOC/liter. Cold mix asphalt produced from cutback solvents requires a higher temperature (55–115 °C) and produces significantly more VOC (300–500 g VOC/liter). Anionic emulsions tend to preferentially bind to siliceous aggregate (such as granite), while cationic emulsions tend to carbonate the aggregate (such as limestone). High viscosity cutbacks (such as Grade 3000) must be heated to at least 80 °C in order to rapidly develop strength [67,71,72].

Once the CMA has been laid down, it is then rolled both statically and vibrationally to produce a density of less than 10% air voids. Curing occurs over approximately 28 days at room temperature; however, this time frame may be reduced through the use of chemical accelerators under cold weather conditions. Storage of cold mix asphalt in insulated silos or under cover at a temperature of 10–30 °C will help preserve its workability and prevent premature emulsion breakage [64,65,66,67,68].

## 5. RAP in CMA: Rejuvenator-Driven Performance and Challenges

Studies on recycled materials in geotechnical fields and pavement projects are prominent in the literature [73,74,75,76,77]. Engineers utilize RAP in CMA to conserve natural resources, reduce project costs, and minimize landfill waste [76,78]. Incorporating RAP also lowers the energy demand of CMA production compared to HMA [79,80]. Laboratory studies confirm that CMA with RAP maintains strong mechanical and durability performance, meeting essential pavement standards [79].

A study by Chegenizadeh [81] demonstrated that the two primary variables, BE content and curing time, played a significant role in the experimental methods. Their research results showed that a mixture containing 4% BE achieved superior results with a resilient modulus of 2510 MPa after 12 weeks, ITS of 561 kPa under dry conditions, and a 49.34% increase in fatigue life compared to mixtures with 2% BE. This mixture also exhibited high resistance to permanent deformation, with a rut depth of only 9 mm. However, it should be noted that Chegenizadeh’s research, conducted in the climate of Western Australia, focused solely on laboratory testing and did not assess the long-term durability of the test samples. In the same manner, Flores [27] investigated compaction conditions for cold-recycled mixtures (CRM) created with 100% RAP and asphalt emulsion and investigated the effects of varying emulsion and cement contents upon the mechanical properties of the mixtures.

**Figure 11:**
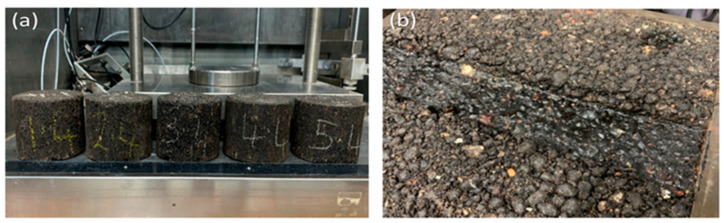
(**a**) Marshall compaction samples (101.6 mm diameter, 63.5 mm height); (**b**) wheel tracking test samples (305 mm × 305 mm × 50 mm) [81].

As shown in Figure 11a,b [81], curing samples were processed under room and oven conditions at 50 °C. The compacted samples were prepared using a gyratory compactor, with an optimum compactive energy of 200 gyrations for samples measuring 100–110 mm and 100 gyrations for samples measuring 60–63 mm. Mixture B, containing 4% emulsion and 2% cement, demonstrated superior mechanical properties, achieving an ITS of 520 kPa, an ITSR of 85%, a permanent deformation rate of 0.08 mm per 1000 cycles, a stiffness modulus of 1800 MPa, and a fatigue life of 200,000 cycles. This clearly indicates the positive effects of cement and emulsion upon the water-resistance, rutting-resistance, and fatigue-resistance characteristics of the mixture. Although both of these studies have provided encouraging data for 100% RAP, other studies suggest mixing up to 35% recycled concrete aggregate (RCA) into binder courses and up to 30% RCA into surface courses to further improve structural performance and environmental sustainability of RAP-based materials [70,73,81]. Rejuvenators, also called recycling agents, are mandated in the production of CMA when RAP is added. These agents restore the properties of the aged RAP binders and increase rock stability and life. Rejuvenators, which could be vegetable oils or crude oil derivatives, will increase the effectiveness of RAP. The application dosage should be from 2% to 20% of the weight of the asphalt binder, depending on the rejuvenator used and the properties of the binder. Their use in application is essential to obtain the best results, which will require prolonged homogenization and mixing [79,80,81,82]. The use of rejuvenators will decrease void content and increase the resistance to water and frost, resulting in a more durable pavement. Rejuvenated RAP binders exhibit superior mechanical properties, including higher fatigue life, improved flexibility, and increased resistance to heavy traffic loads. Laboratory studies [61,81] have demonstrated the efficacy of several rejuvenators derived from vegetable oils, such as rapeseed oil, linseed oil, waste vegetable frying oil, and combinations of rapeseed oil and waste frying oil mixed with soft bitumen. These rejuvenators have effectively reactivated the aged binder in RAP [82,83,84]. Studies conducted by the authors, which consisted of field trials for three years at secondary road locations, indicated that pavements utilizing rejuvenators made from vegetable oils did not sustain any damage during this time frame and, therefore, enhanced both the durability and sustainability of the pavement material [83,84].

## 6. Mix Design Approaches for CMA

### 6.1. Overview of CMA Mix Design Philosophy

CMA has some difficulties when it comes to designing mixes, especially because the binder used (bitumen emulsion or cutback) will be liquid at room temperature; therefore, no heat is required to mix the materials. CMA is generally made up of very large quantities of recycled asphalt products (RAPs), generally between 90% and 100%, which are sustainable and energy-efficient paving materials [27,39,61]. A successful CMA is dependent on several items being taken into consideration: gradations of the aggregates; quality of the binder; curing time; and temperature. The absence of a universal mix design method for CMA has led to various approaches, each emphasizing different aspects of the mix.

This section provides an overview of the key CMA mix design methods, comparing laboratory procedures, standard specifications, performance-based approaches, and optimization techniques. Each method aims to improve the durability and performance of CMA in real-world applications

### 6.2. Laboratory-Based Mix Design

#### Key Factors Affecting Laboratory CMA Performance

The CMA Laboratory Mix Design Process is very complex, and there is no single method. That is universally accepted; however, most laboratories use the Marshall method to assist with designing a CMA. In contrast to HMA, CMA utilizes a bitumen emulsion or cutback as a binding agent, which remains a liquid at room temperature; therefore, it is not necessary to heat the mixture during manufacturing [75,76,77,78,79]. Several factors affect the mechanical properties and durability of CMA, including aggregate grading, type and quantity of binder utilized, binder quality, moisture content, voids ratio, curing temperature, curing time, and presence of filler materials [80,81,82].

One of the major challenges in CMA mix design is reproducing actual field curing conditions within laboratory settings. To address this difficulty, South Africa introduced foamed asphalt technology as an alternative approach to developing a more standardized CMA design methodology [85]. Research indicates that foamed asphalt mixtures often exhibit performance comparable to conventional emulsion-based CMA in road applications. Here is a more natural, non-AI-sounding version. Numerous laboratory studies have explored accelerated curing conditions—from room temperature up to 60 °C—to better represent field behavior. Table 1 provides an overview of the curing protocols commonly used in previous research.

Aggregate selection also plays a critical role in CMA performance, particularly when choosing the appropriate type of asphalt emulsion. Emulsions are classified as cationic or anionic depending on the surface charge characteristics of the aggregate.

High SiO_2_ content in aggregate produces a highly negative charge to the surface of the aggregate; this increased negativity enhances the bonding properties of cationic asphalt emulsions, creating an enhanced, durable bond to improve the durability of the final CMA product. The interaction of the asphalt emulsion and the aggregate is critical to the long-term performance of CMA, as indicated in references [34,86,87,88]. There is currently no universally accepted standard method of developing CMA designs, but virtually all CMA research has focused on using the mix design process developed by AI MS-14 [65,72].

**Table 1:** Curing of CMA and different bitumen types, which have been adopted in various studies.

References	Conditioning Temperature	Time for Conditioning (Days)	Bitumen Type
[89]	38 °C	7, 28	BM
	Ambient	1	FM
	38 °C	7–14	BM
[85]	Ambient	7	FM
	Ambient	28	FM
	60 °C	2	FM
[90]	60 °C	1, 3, and 7	Both
[91]	60 °C	2	BM
[92]	40 °C	18–21	BM
[93]	60 °C	2	Both
	20 °C	101	BM
[94]	38 °C, 40 °C, 60 °C	1, 7, and 28	BM
[81]	Ambient, 40 °C, 60 °C	7–84 days (1–12 weeks)	CSS
[95]	25 °C, 40 °C	28 days	Foamed 50/70 bitumen

BM—Bituminous Mixture; FM—Foamed Mixture; CSS—cationic slow-setting emulsion, aggregate gradation, and moisture resistance. The goal is to optimize CMA performance under various conditions, ensuring the construction of long-lasting, durable pavements.

To provide a broader perspective, Table 2 provides a side-by-side comparison of the mix design methods for CMA, as outlined in different standards, including AI MS-14, TG [96], IRC: SP:100 [97,98], and AASHTO PP 80-20 [99]. Each standard presents its approach to determining the appropriate mix based on traffic load, aggregate gradation, and moisture resistance. The goal is to optimize CMA performance under various conditions, ensuring the construction of long-lasting, durable pavements. Therefore, the interaction between the asphalt emulsion and aggregates is essential for the long-term performance of CMA [86,87,88]. Currently, there is no widely acknowledged standardized procedure for designing CMA. However, most of the research focuses on the mixed design process established by AI MS 14 [65,72].

### 6.3. Asphalt Institute Method of Mix Design

The Asphalt Institute method for designing CMA is a well-documented approach. Originally formalized in the Basic Asphalt Emulsion Manual (AEMA/Asphalt Institute, 1979 [37]) and the Asphalt Institute [65]. It includes both the Hveem method and the Marshall mix design, though the Marshall approach has become dominant due to its wider acceptance by agencies such as the Illinois DOT.

The Hveem method gained popularity in California. The Illinois Department of Transportation created the Marshall mix design and, subsequently, the Asphalt Institute released Manual Series No. 14 (AI MS-14) and later MS-19. AI MS-14 became the more widely utilized method for CMA mix design. The AI MS-14 procedure features several essential elements. These elements influence the mix design [65,72]. This technique highlights the significance of aggregate gradation, suggesting the use of either dense or gap-graded aggregates to guarantee effective compaction and load-bearing capacity. The IEC is determined using two empirical methods. Parameter P denotes the percentage of initial residual asphalt content (IRAC) relative to the total mass of the mixture, as shown in Equation (1).

(1) $$P=\left(0.05A+0.1B+0.5C\right)\times 0.71$$

Here, A is the percentage of aggregate retained on a sieve of 2.36 mm. B is the percentage of aggregate passing a sieve of 2.36 mm but retained on a sieve of 0.075 mm. C is the percentage of aggregate passing a sieve of 0.075 mm.

When P has been determined, the IEC is to be calculated using Equation (2).

(2) $$IEC=\left(\frac{P}{X}\right)\%$$

Here, X is a factor that represents the emulsion binder content.

The initial IRAC can be determined with empirical formulas that are based on sieve sizes (breakpoints) at 2.36 mm and 0.075 mm to calculate an IEC as well. There is a minimum coating value of 50% that indicates sufficient coverage of aggregates. The optimum total liquid content (OTLC) is established with the moisture content that produces the greatest possible dry density within the mix.

The curing procedure is typically performed in four stages:

1. 24 h in mold at 25 °C;
2. 24 h in oven at 40 °C;
3. 24 h stabilization in mold at 25 °C;
4. 48 h water immersion (soaked stability evaluation).

A revised method eliminates OTLC-driven compaction, recommending instead that mixtures be air-dried to a workable state before compaction [65,72,98]. Even though it is widely accepted, there are certain limitations to the acceptance of AI MS-14.

- It does not specify acceptable porosity ranges.
- The definitions of fully cured and ultimate strength remain ambiguous.
- Moisture parameters used in volumetric analysis are inconsistent with modern standards.

These limitations have prompted agencies to develop modified variants tailored to local materials and climate conditions.

### 6.4. Performance-Based Mix Design

Although the Marshall test remains widely used for assessing binder content and mix composition, it does not capture fatigue resistance, permanent deformation behavior, or long-term performance. Thus, the CMA mix design is being moved to performance-based methods by contemporary developments in CMA mix design. A performance-based method of the above type is shown in the Asphalt Academy TG 2 Guidelines [96], which has classified the mix design into three categories with respect to traffic loading intensity.

- Low traffic (<3 MESA): ITS (dry/wet) and TSR;
- Medium traffic (3–6 MESA): ITS after moisture equilibration and soaking;
- High traffic (>6 MESA): triaxial testing for cohesion, friction angle, and moisture durability.

The modified AASHTO compaction method can be utilized to obtain the best moisture conditions for the production of triaxial test specimens using vibratory compaction and split molds [100]. The use of performance-based testing will result in a reduction in cost both economically and environmentally, as less asphalt is required in comparison to traditional methods of testing, particularly if reclaimed asphalt pavement RAP is included in the testing process [100]. Cement, fly ash (FA), and geopolymers are additives that have been found to improve the mechanical properties (i.e., strength, moisture resistance, and durability) of chemically modified asphalts (CMAs). Cement and hydrated lime have been found to provide an improvement in indirect tensile strength (ITS), while fly ash (FA) and geopolymers have provided improvements in moisture resistance [101]. However, extended curing time has shown to produce improved results (up to 30 days), and challenges still exist in simulating field curing, determining long-term performance, and assessing the environmental impact of additives. Nevertheless, CMA continues to be a suitable, cost-effective, and sustainable alternative to hot mix asphalt (HMA) [96,101].

#### 6.4.1. The Phenomenon of TSR Values Above 100%

A TSR of 100% or less is commonly found in conventional asphalt mixes due to its measurement of moisture sensitivity by comparing the ITS of wet-conditioned samples to those that are dry-conditioned. A number of recent investigations of cement-modified asphalt (CMA), however, report TSR values to be over 100%. This finding does not represent an error of measurement but rather an indication of continued cement hydration after exposure to wet conditions for some time [45,102,103,104,105,106,107,108,109,110,111].

In CMA mixtures, the dry condition specimens may have incomplete hydration occurring at the time of testing. Upon addition of water during the wet conditioning process, this hydration process can continue, resulting in the formation of further calcium-silicate-hydrate (C-S-H) gel. The addition of this gel into micro-voids enhances the internal bond between aggregate particles within the internal matrix of the mixture, resulting in increased ITS values for wet-conditioned specimens compared to those of dry-conditioned specimens.

**Figure 12:**
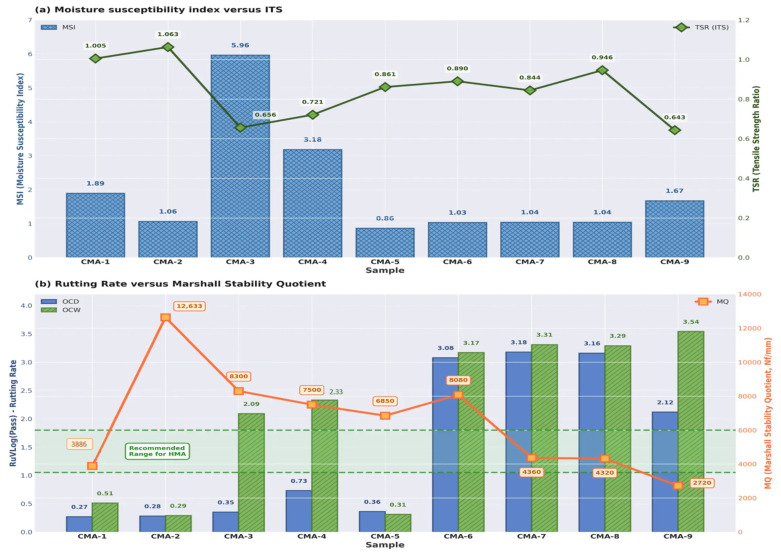
(**a**) Moisture susceptibility index versus ITS; (**b**) Rutting Rate versus Marshall Stability Quotient [2].

A compelling example of this comes from a study by M. Rezaei [2] on a CMA mixture containing 2% Portland cement recorded an impressive TSR of 106% (as illustrated in Figure 12a,b). The strength of the wet specimen was actually higher than the dry one. The researchers explained that this was not a case of water causing damage; instead, the immersion triggered a secondary curing phase that boosted the material’s overall strength and resilience. Therefore, when we see a TSR value over 100% in a cement-modified mix, we should not view it as a mistake. Rather, it should be seen as a sign of a well-designed, high-performance mixture. It tells us that the cement is continuing to hydrate and cure, creating a denser and more water-resistant final product. This characteristic is a significant contributor to the exceptional durability and moisture resistance we see in modern cold mix asphalt designs [2,102,105].

#### 6.4.2. Optimization of CMA Mix Design Using Response Surface Methodology (RSM)

To further refine these designs, the use of RSM has, in recent years, enhanced the optimization of CMA by statistically modeling the interactions between the mix design parameters. This advanced statistical approach was employed when Al-Jumaili [112] investigated the effect of three key experimental parameters (X): aggregate emulsion content (AEC), pre-wetting content (PWC), and compaction temperature (CT). Their study used a quadratic regression model (Equation (3)) to relate the response variables (Y)—including mechanical properties (ITS and ITSM) and volumetric properties (air voids and dry density)—to these parameters.

(3) $$Y=\beta^{0}+\sum_{j=1}^{k}\;\beta_{j}X_{j}+\sum_{j=1}^{k}\;\beta_{jj}X_{j}^{2}+\sum_{i<j,j=2}^{k}\;\beta_{ij}X_{i}X_{j}+e_{i}$$

where Y denotes the response variable, X_i_ and X_j_ are experimental parameters, β are regression coefficients, k represents the number of factors, and e is the random error.

Utilizing the Box–Behnken Design, the number of test runs was efficiently reduced from 225 to 24, while maintaining a high predictive capability (R^2^ > 0.98). The model was used to optimize polymer type (PET/PE) and amount (0–20%) over a 30-day testing period, identifying 20% PE after 30 days as the optimal combination, yielding the highest stability (42.98 kN) and quotient (8.66 kN/mm). Therefore, RSM is a viable method to develop a sustainable approach to optimize CMA composition, improving both mechanical properties and circular economy goals [112,113].

A summary of various CMA performance-based mix design approaches is provided in Table 2, namely, AI MS-14 [65,72] along with TG 2 [96], AASHTO PP 80-20 [99], and IRC: SP:100 [97,98]. Of these, TG 2 uses an engineering-oriented methodology that defines the type of mixture according to traffic loads and emphasizes ITS and triaxial tests as measures of durability. The empirical nature of AI MS-14, AASHTO PP 80-20, and IRC: SP:100 means their primary focus is upon density, stability, and coating characteristics rather than evaluating long-term performance.

## 7. Performance Evaluation of CMA

CMA presents a sustainable option for modern road construction by incorporating a high volume of RAP (90–100%) and achieving a 40–50% reduction in energy usage compared to conventional HMA. Although traditional HMA tests, such as MS, ITS, and permanent deformation resistance, remain relevant for CMA, their effectiveness depends on several factors, including curing duration, ITS, TSR, aggregate gradation, binder type, emulsion formulation, and air void content. Other aspects, such as mix design parameters (filler composition) and environmental conditions, also affect performance. These essential performance indicators, derived from numerous studies, are comprehensively outlined in the accompanying Table 3.

### 7.1. Laboratory Studies on the Performance of CMA

#### 7.1.1. Effect of Aggregate and Gradation on the Performance of CMA

The properties of CMA are primarily determined by the type, quality, size, and origin of aggregates, which form about 95% of the mixture skeleton [115,116]. Aggregate chemistry and surface characteristics influence adhesion to asphalt binders, with alkaline aggregates (e.g., limestone) offering better adhesion and water resistance than acidic ones (e.g., basalt or granite) due to higher surface free energy [115,117]. Surface charge also affects compatibility: negatively charged silicates bond well with cationic emulsions, whereas carbonates suit anionic emulsions [118,119].

Grading significantly impacts density and mechanical performance. Dense-graded (DG) mixes provide higher Marshall stability (MS), while gap-graded or open-graded (OG) mixes enhance indirect tensile strength (ITS) and creep through the stone-on-stone effect [102]. DG CMAs outperform OG mixes in permanent deformation resistance (*p* = 4.65 × 10^−6^) due to a denser aggregate skeleton and lower air voids. Performance correlations vary by grading: DG mixes depend on dust-to-binder ratio (R^2^ = 0.952), OG mixes on coarse aggregate content (R^2^ = 0.897), and bitumen content shows a negligible effect (*p* = 0.993), highlighting grading as the dominant factor.

Systematic gradation design, such as the Bailey method, ensures optimal mix behavior, with Dense Bitumen Macadam (DBM) showing greater stability and durability than Semi-Dense Bituminous Concrete (SDBC), while gap-graded SMA offers sustainability benefits [102,103]. Incorporating recycled aggregates (RAP, RCA) can improve ITS by 12.8%, cost, availability, and thermal stability, provided segregation, moisture control, and binder–aggregate interactions are carefully managed [107,116].

#### 7.1.2. Effect of Additives on the Performance of CMA

Additives incorporated through dry or wet processes (pre-blended into the bituminous emulsion) are crucial for performance enhancement of CMA. The most extensively studied and used is cement. Its large surface area and negative surface charge enhance the breaking of the emulsion rapidly by raising the pH in the aqueous phase, inducing hydration reactions yielding significantly enhanced mechanical strength [25,102]. Adding 1% OPC has been shown to increase MS by 250–300% compared to unmodified CMA. Higher dosages, such as 1–2% of rapid-setting cement, accelerate early strength gain. Cement dosages in the range of 1.5–2% enhance MS by 210–280%, attributed to pozzolanic reactions (R^2^ = 0.91, *p* < 0.001) [25,105]. Finer cement particles (FCPs) improve the microhardness at the aggregate–cement interface and strengthen the bond between aggregates and emulsion, leading to improvements in resilient modulus, moisture resistance, and rutting resistance [25,108]. FCPs enhance the microhardness of the aggregate–cement mortar interface, and cement addition strengthens aggregate–emulsion bonding, improving resilient modulus, moisture resistance, and permanent deformation resistance [108,109]. Fully cured CMA with 1–2% OPC has, in some cases, exceeded the mechanical performance of conventional HMA [25,108]. Further enhancement is observed with ternary cementitious blends. A combination of 2% OPC, 1% FA, and 1% ground GGBS increased ITS by 20%, residual stability to 95%, and permanent deformation resistance by a factor of 10. This was attributed to a denser microstructure and increased formation of calcium-silicate-hydrate (C-S-H) products [45]. The blend showed a bending creep stiffness of 2900 MPa, which is 1.1 times greater than that of CMA containing only 2% OPC. The advantage of high-temperature stiffness was, of course, gained at the expense of low-temperature flexibility, resulting in a 15% decrease in failure strain. Dynamic stability increased to 52,751 cycles/mm, indicating excellent resistance to deformation [45]. The performance of the systems based on geopolymer binders is also good.

A blend consisting of 4% GGBS, 2% calcium carbide residue (CCR), and a waste alkaline solution of calcium (GCAE) gave an ITSM of 2465 MPa after only three days of curing. This value was 13 times higher than that of traditional limestone filler (TLF) mixes and exceeded the 28-day stiffness of conventional HMA by 110% [35]. Rut depth under 10,000-wheel tracking cycles decreased from 9 mm (TLF) to 1.7 mm (GCAE), outperforming HMA by a factor of 2.5–4. The GCAE mix also improved water resistance, as evidenced by a rise in the stiffness modulus ratio (SMR) from 75% to 103%. Low-temperature cracking resistance increased by 9%, with fracture toughness improving from 7325 to 7985 N/mm^2^ [35]. Other mineral additives offer further performance enhancements. For example,

- Silica fume (1% by aggregate weight) increased stiffness to 72.2 MPa [120];
- Type C FA (3–11%) enhanced fatigue life and moisture resistance, though doses above 15% induced brittleness [110];
- Hydrated lime slurry (2%) improved resilient modulus by 32.46% and reduced rut depth by 58%, outperforming lime powder [109,110,111].

#### 7.1.3. Effect of Fillers on the Performance of CMA

Fillers, which are the smallest pieces of material used to make an asphalt mixture, greatly influence how well a Compacted Mastic Asphalt (CMA) performs with regard to its stiffness, water resistance, and deformation resistance [36]. The way that fillers enhance the bonding between the aggregates and the binder is through the process of filling voids, preventing water from entering these voids, and creating areas where the weight of traffic can be applied. Fillers that are very porous will have poor performance. Other important characteristics of fillers include a narrow gradation size, low amounts of clay, hydrophobic materials, high calcium carbonate content, and a hardening effect caused by Ca(OH)_2_ [121,122]. Using manufactured binary (cement + fly ash (FA) or ground granulated blast furnace slag (GGBS)) and ternary filler blends will increase the creep stiffness of the mixture and lower the amount of permanent deformation [46,105,123]. Using cement at 2.75–5.5% or using Ordinary Portland Cement (OPC) at 0–6% will increase the structural properties of the mixture. Using FA at 0–6% and using GGBS at 1–3% will also increase the stiffness of the mixture and the amount of water resistance because of their pozzolanic reaction. Alkali-activated fillers, such as high-calcium fly ash or catalytic residue, also enhance mechanical strength, moisture resistance, and thermal stability.

Latex-modified CSS-1hL emulsions significantly improved moisture resistance, particularly for granite aggregates (CR 96.7%, BR 97.5%, and TSR 97%), outperforming unmodified CSS-1h and limestone-CSS-1hL mixes, while high-viscosity CSS-2 emulsions were less effective. Strong correlations (R^2^ = 0.91–0.99) between CR, BR, and TSR validated the modified boiling test for moisture sensitivity, and higher emulsion content reduced ITS at 12% air voids, highlighting the importance of dosage [124].

SEM studies [105] showed that silica fume (SF, <1 µm) reduced air voids to 8.5–10.3% and minimized stiffness loss at 40 °C (28% vs. 95% for limestone), by stimulating nano-scale hydration and improving early stiffness 3–17 times within seven days. GGBS enhanced mechanical interlocking and moisture retention, achieving the highest creep stiffness (282.1 MPa) and ITSR >85%, while FA alone delayed hydration. Ternary blends (SF + FA + cement or GGBS) produced dense C-S-H gels, reducing moisture intrusion and axial strain by 94–95%, balancing early strength, high-temperature stiffness retention (72% at 40 °C), and long-term durability.

A key to sustaining the use of reclaimed asphalt pavement is finding ways to reduce the negative effects that occur as a result of using reclaimed material. Researchers have found that the addition of limestone to the reclaimed mix provides many benefits, including increased stability, improved tensile strength and resiliency, as well as decreased permanent deformation. The absence of this additive may be detrimental to the durability of the mix. As an example, researchers tested the properties of 100% reclaimed asphalt pavement mixes with and without additives. They concluded that the presence of additives significantly reduced raveling (39.3% Cantabro loss) and air void content (10.3%) but had no significant effect on the susceptibility to cracking (CT Index = 77.9). They also noted that the inclusion of 100% reclaimed asphalt pavement into the mix resulted in a 49.3 ± 5.1% (*p* = 0.0032) increase in fatigue life [102,105].

#### 7.1.4. Effect of Fiber Addition on the Performance of CMA

Fibers added to CMA have greatly improved the physical and durability properties of CMA, such as high-temperature resistance, cracking resistance, and moisture resistance and thus can be used as an alternative for HMA. The investigation into the performance of CMA using natural, synthetic, and recycled fibers has been carried out. As an example, Zhu and Xu [106] conducted research on polyester fibers (PFs) and basalt fibers (BFs). The authors reported that PFs and BFs were able to improve the mechanical and durability characteristics of CMA.

BFs increased Marshall stability (MS) by 44.9% at 28 days, whereas PFs improved MS by 32.8%. Conversely, PFs demonstrated 1.5 times greater flexural tensile strain compared to conventional emulsified asphalt mixtures (CEAMs) due to their longer fiber configuration. ITS increased by 39.4% and 31.7% for BFs and PFs, respectively, attributed to their three-dimensional interlocking with the cement–asphalt matrix. Regarding durability, BFs exhibited the highest raveling resistance, followed by PFs (13.6%) and CEAM (18.2%). PFs also achieved the highest dynamic stability at elevated temperatures (33,116 passes/mm), slightly exceeding BFs (31,460 passes/mm).

**Figure 13:**
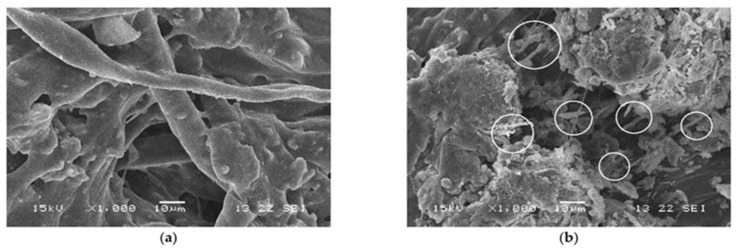
(**a**) SEM micrographs of fiber-reinforced cement-emulsified asphalt mixtures (FRCEAMs); (**b**) FRCEAM-PF (polyester fiber) and FRCEAM-BF (brucite fiber) [106].

SEM images (Figure 13a,b) revealed that PFs bridged crack openings due to their thin and elongated morphology, whereas BFs, varying in length (0.2–4 mm) and diameter (2–4 μm), formed an extensive network that improved void reduction and load transfer. Similar benefits of polymer fibers in HMA have been reported by Bocci [123], including enhanced tensile strength and reduced low-temperature cracking. Polypropylene fibers have been shown to reduce flow values and improve CMA stability; however, excessive content can negatively affect overall performance [106,125]. Fibers act primarily by absorbing fracture energy and arresting crack propagation [123,125]. Moreover, combining fibers with polymeric additives such as acrylic provides further enhancement in crack resistance and mechanical strength in CBEM, as demonstrated by Al-Kafaji [126]. The behavior of the CMA depends on the fiber type, length, and percentage added to the CMA. Optimal performance was obtained for polypropylene fibers at a length of 40 mm and percentages of 0.1–0.25%. At higher percentages of polypropylene fibers, the stability of the CMA is reduced [127]. The addition of aramid and PET fibers to the CMA improves the rutting resistance, CT Index, and fracture energy of the CMA. These improvements were achieved by adding 0.065–0.1% aramid or PET fibers to the CMA when the asphalt content was 5.5% [106]. The natural fibers (hemp, jute, and coir) used to improve the stiffness and crack resistance of the CMA have optimal performance levels of approximately 0.35% fiber content and lengths of 14–15 mm [125,128,129,130]. FEM studies also support the trends observed in the laboratory studies [128,129]. In summary, despite being less studied than HMA, optimized fibers can significantly improve the rutting resistance, fatigue life, and deformation properties of CMAs [125,127,128,130].

#### 7.1.5. Effect of Compaction on the Performance of CMA

Compaction critically affects the mechanical performance and durability of CMA by influencing air voids, aggregate interlock, and binder distribution, which are essential for structural integrity under traffic loads. In the laboratory, Marshall compaction (typically 50 blows per face) is commonly employed. P. Deb [120] reported that increasing blows from 50 to 75 slightly improved Marshall stability (MS) and reduced air voids, though excessive energy caused aggregate fracturing and binder migration. The gyratory compactor (GC), providing kneading and rotational shear, is better suited for non-cohesive wet mixtures, allowing more uniform densification compared to the impact Marshall method [33,131]. Yang et al. [33] evaluated cold-recycled CMAE with RAP, asphalt emulsion, cement (0–2%), and limestone under three compaction strategies (50, 75, and double compaction) and two curing regimes. Double compaction reduced voids by 15–20%, increased ITS by 28% (1202.7 vs. 943.1 kPa), decreased rut depth (12.0 → 7.5 mm), and improved CSED by 40–50%. However, excessive compaction in cement-rich mixes increased brittleness, reducing failure strain by 15%. Calibration studies by Dulaimi [132] showed equivalence between Marshall and Superpave protocols (50 Marshall blows ≈ 80 SGC gyrations; 75 blows ≈ 120 gyrations), indicating that reduced binder content in Superpave mixtures can maintain target density while reducing rutting and bleeding.

**Figure 14:**
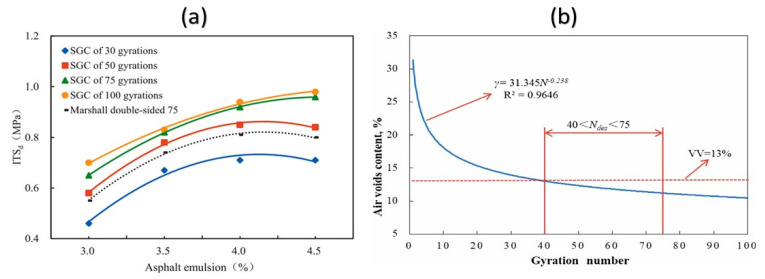
(**a**) ITS of CRM under various compaction methods; (**b**) GC curve for CRME [134].

Further increases in gyrations (75–100) produced minimal ITS gains (0.93–0.98 MPa), confirming 50 gyrations as the optimal compaction level for this mix, as shown in Figure 14a,b. Air void content critically influences CMA performance. Using SGC, target air voids of 6%, 8%, 10%, and 12% were achieved in mixtures containing 60% RAP [133]. Higher compaction (120 gyrations for 6% voids) reduced voids and enhanced performance: MIST-TSR remained above 80% for ≤8% voids, while rut depth stayed below 12.5 mm. Insufficient compaction (30 gyrations for 12% voids) increased voids, lowering MIST-TSR to 72% and accelerating rutting to failure levels. Stripping resistance also deteriorated, with inflection points decreasing from 8280 passes (8% voids) to 2680 passes (12% voids). Under-compaction reduced MS (4.2 → 2.6 kN), decreased fracture energy by 21.5%, and increased mass loss from 6.5% to 12.3%. Optimal compaction (<8% voids) balances structural density and molecular cohesion, whereas poor compaction (>10% voids) promotes material deterioration [83,133].

Densification curves indicate that 80–120 gyrations produce densities equivalent to 50–75 Marshall blows, confirming the kneading efficiency of GC. Most mixes reach a locking point between 40 and 75 gyrations; beyond this, further effort yields negligible densification, reflecting the maximum attainable density for a given gradation. AASHTO R-35 [135] correlates laboratory compaction to traffic loading, recommending 50 gyrations for light traffic (<0.3 million ESALs) and 125 gyrations for heavy traffic (>30 million ESALs). Field studies corroborate laboratory findings: GC-compacted CMA exhibited only 0.9 mm rutting after 10 years, indicating enhanced durability [5,7,77]. Early compaction is crucial, as emulsion mixes lose workability after 24 h, especially when cement is included. Air voids of 5–10% may require increased compactive effort, but modern methods allow CMA to be effectively placed on high-traffic roads, consistent with AASHTO R-35 specifications [7,9,10,136].

#### 7.1.6. Effect of Curing on the Performance of CMA

There have been many studies on the curing process of the asphalt emulsion CMA, because curing is an important step for improving the mechanical properties of CMA over time. The curing process determines when emulsion breakage will occur, how much water will evaporate from the mixture, and how hydrated the additives will be in the mixture; therefore, it is the governing mechanism that determines the rate at which CMA will develop strength and stiffness. However, researchers were concerned about CMA’s low early strength and lengthy curing times; so, they looked into the use of additional materials (additives), like Portland cement, FA, and lime, to reduce curing times and improve overall performance. One study [49] identified a three-stage evolution in the mechanical properties of CMA containing 0–6% OPC, as shown in Table 4.

Strong linear relationships were confirmed among MS, ITS, and ITSM for all cement dosages (R^2^ > 0.85) [49]. Another study [81] explored how curing time (1–12 weeks) and (BE) content (2–4%) affected the mechanical performance of CMA made with 100% RA. Specimens were compacted at the optimum moisture content (5.1%) and cured for 1–12 weeks under dry and soaked conditions.

Key findings were as follows:

- Curing time: Extending the curing period from 1 to 12 weeks raised the resilient modulus by 195% (0.85 → 2.51 GPa) and ITS by 144% (230 → 561 kPa) for the 4% BE mix.
- Moisture: Soaked curing reduced ITS by 14%, demonstrating moisture sensitivity.
- Emulsion dosage: Increased BE from 2% to 4% and cut rut depth from 13.6 to 9.0 mm and lengthened fatigue life by 49% [137].

Although the 4% BE mix cured for 12 weeks delivered the best laboratory performance, the authors noted that Western Australian climate conditions and the long curing duration may limit direct field extrapolation. CMA performance also depends on temperature–time history [138].

Fatigue life decreased with prolonged curing (due to moisture loss and embrittlement) but improved when the curing temperature rose from 5 °C to 50 °C, owing to faster binder–aggregate adhesion. To quantify these effects, Chelelgo et al. [138] defined a maturity index M:

(4) $$M=\sum \alpha \Delta t\cdot T\alpha =eB\left(T-Tr\right)$$

where Tr: temperature, B: temperature sensitivity factor (1/°C), T: cure temperature (°C), and Δt: cure duration (days). Two predictive models, parabolic and linear hyperbolic functions, were developed to relate fatigue strength (Nf) to maturity (Equation (5)).

(5) $$N_{f}=N_{fu}\times \left(1+\sqrt{kM}\right)\sqrt{k}$$

where Nf: fatigue strength at a given maturity, Nfu: ultimate fatigue strength, k: curve-fitting constant, and M: maturity index.

The model achieved R^2^ > 0.80 for strain levels of 125–200 µmm^−1^ and outperformed a linear variant (R^2^ < 0.70 for strains > 200 µmm^−1^). A parallel Arrhenius analysis yielded the activation energy, as shown in Equation (6).

(6) $$Ea=B\times R\times {\left(T_{r}+273\right)}^{2}$$

Ea varied between 29.4 kJ/mole at a 125 micro m/m strain level and 50.6 kJ/mole at a 250 micro m/m strain level, consistent with typical values for bitumen. At higher strain levels, Ea increased, and therefore, the relationship was more sensitive to temperature as well as the mechanical load. The parabola model continued to be reliable, while the linear model became less accurate at strains greater than 200 micro m/m (R^2^ < 0.7). Including the curing history (time × temperature) improved the fatigue strength predictions, thereby providing support for the implementation of maturity-based protocols to improve the durability of CMA [138].

### 7.2. Field Validation and Long-Term Performance of CMA

Although laboratory testing of CMA is crucial for evaluating mechanical properties, field validation is essential to confirm long-term performance and real-world applicability. Several studies have highlighted the potential of CMA technologies for sustainable pavement construction under varying climate and traffic conditions.

For instance, Wu Shenghua [14] studied a 100% RAP cold mix pavement on a low-volume road in Florida, USA. He found that the mixture he created using a rejuvenator to restore the aged binder had very little to no cracking or rutting after 34 months of being placed into service; however, he did find some evidence of weathering. It also stated that same-day compaction was necessary to obtain the best possible in-place density.

Dulaimi [35] evaluated a geopolymer-based Cold Asphalt Emulsion (GCAE) mixture and found it had 13 times greater early age strength (after 3 days) and significantly better rutting resistance (1.7 mm vs. 9 mm for a control mixture). According to SEM results, this high mechanical performance was due to a dense matrix developed through geopolymer technology. Therefore, geopolymer technology may be able to improve upon the poor performance of many CMAs.

In a similar study, Gu Fan [21] conducted a series of lab and field tests of a central-plant recycled cold mix with foamed bitumen (CCPRF), in comparison to HMA and CIR. Lab tests indicated that the dynamic modulus for CCPRF was less than that for HMA; however, the fatigue life and rutting resistance of CCPRF were comparable to those for HMA. In addition, field observations supported the viability of CCPRF in real-world conditions, and CCPRF performed better than both CIR and other types of mixes.

To provide a comprehensive overview of real-world performance, Table 5 summarizes key field trials across diverse global conditions. Based on these trials, it is evident that modern CMA can perform at least equally well as conventional asphalt in many demanding applications:

- Exceptional Durability under Heavy Traffic: On Scotland’s A90 trunk road, the Tayset CMA (70% RAP) exhibited no signs of distress after 10 years of service under extremely high traffic loads (>10 M ESA). Additionally, the stiffness stabilized at a very high level of 6 GPa within 6 months, demonstrating the long-term durability of the CMA, as well as significant carbon savings [139].
- Resilience in Extreme Climates: In a 15-year study in Sweden, it was demonstrated that CMA is a durable option, with the mix developing few cracks and no rutting over an extreme temperature range of −35 °C to 60 °C [140]. Conversely, field trials in China showed that, while AC-graded patches deteriorated rapidly in extreme cold winter conditions, open-graded LB patches did not develop defects after one year, demonstrating the importance of mix design for cold climates [141].
- Performance in High-Rainfall/High-Traffic Conditions: A CRM-E mix in Malaysia (100% RAP) displayed superior performance under extremely high rainfall and traffic (12,000 VPD/Lane). It produced higher stiffness levels than HMA (28–68%) and demonstrated excellent moisture resistance (TSR = 85–93%) and less than 2.5 mm of rutting after 12 months [142].

**Table 5:** Field performance of CMA.

Authors	Country	CMA Type	Climate and Traffic	Monitoring Duration	Summary of CMA Performance
Shenghua Wu, Cade Marty [14]	USA	100% RAP cold mix (with rejuvenator)	Florida (subtropical); low-volume road	22 months	The 100% RAP CMA exhibited only minor weathering and raveling after 3 years. No cracking or rutting observed. Same-day compaction recommended for improved density and reduced raveling.
J. Yi et al. [141]	China	Solvent-based liquid asphalt with limestone aggregates	Severe winter; medium traffic	10 days–1 year	Field trials revealed that AC-graded cold mix patches failed within a month, whereas open-graded LB patches showed <30 mm deformation and remained intact after one year. The LB mix’s coarser skeleton and higher voids enabled faster curing and improved durability, making it more suitable for winter pothole repair.
Jin, Dongzhao et al. [143]	USA (Michigan)	Cold in-place recycling (CIR)	Cold, wet; low-volume road	20-year modeled life	CIR improved cracking and fatigue resistance under freeze–thaw cycles. Predicted rutting and IRI increases remained minimal, validating CIR for the cold, wet region.
David Allain et al. [144]	USA	CIR	Subtropical (Medium)	N/A	CIR and full-depth reclamation enhanced the structural strength and durability of CMA pavements.
Charmot et al. [142]	Malaysia	CRM-E (100% RAP + 3.5% emulsion + 1.5% OPC; HMA overlay	High rainfall; warm; 12,000 vpd/lane	12 months	CRM-E (100% RAP with emulsion and cement) performed exceptionally under high rainfall and traffic, showing 28–68% higher stiffness than HMA, strong moisture resistance (TSR 85–93%), minimal rutting (<2.5 mm), and no cracking after 12 months. A same-day HMA overlay further improved early strength without affecting long-term durability.
S. Kolo et al. [61]	Nigeria	DPWS-modified (Dissolved Polythene Waste Sachets) bitumen	Tropical/subtropical; urban traffic	4 months (intensive field monitoring)	LB-graded CMA performed well in cold regions (<30 mm deformation/year), while AC-graded mixes failed early. In tropical climates, DPWS-modified CMA with recycled polythene showed higher strength and minimal settlement, emphasizing the value of CMA-specific standards and recycled materials.
Dennis Day et al. [139]	UK	Tayset CMA (70% RAP + 30% virgin aggregate) with C60B5 emulsion	Cold, damp; >10 million ESA	10 years (2008–2018)	The Tayset CMA (70% RAP, 30% virgin aggregate, C60B5 emulsion) showed no distress after 10 years and 10 million ESAs on Scotland’s A90. Its stiffness stabilized at 6 GPa within six months, with strong rutting resistance and 43 t CO_2_ savings, confirming its long-term durability and environmental benefits.
Suda, J et al. [140]	Sweden	Cold bituminous emulsion mixture	Tropical/Sub-Tropical	15 years	After 15 years (−35 °C to 60 °C range), CMA displayed few cracks, slow binder ageing, and no rutting. RAP sections outperformed conventional soft asphalt. CMA is validated as an eco-friendly, durable option.

Collectively, these studies evaluate the performance of CMA mixtures containing various additives, rejuvenators, and modifiers in real-world conditions, demonstrating that CMA is a robust and sustainable alternative for a wide variety of traffic and environmental conditions.

## 8. Environmental and Economic Impact of CMA

**Figure 15:**
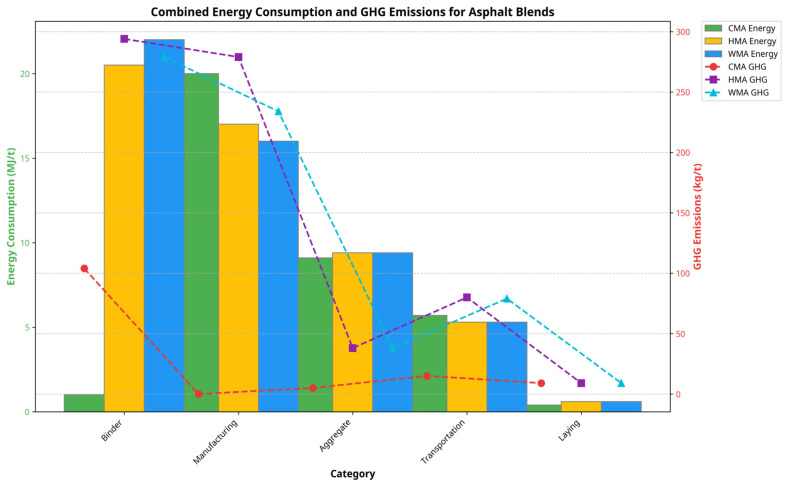
Combine energy consumption and GHG emissions for asphalt blends [146].

The worldwide road industry is a significant contributor to GHG emissions, accounting for about 7–8% of global CO_2_ emissions throughout construction, maintenance, and operational life cycles [117]. HMA is the most energy-intensive, primarily due to the high production temperatures (160–180 °C) required to heat aggregates and bitumen. This process consumes an average of 279 MJ per ton of mix and emits 13.95 ± 6 kg CO_2_e per ton [N = 18], excluding transport and upstream emissions [123,145]. By comparison, WMA reduces energy consumption by approximately 16% (to 234 MJ/t) and CO_2_ emissions to 11.7 kg CO_2_e/t. In contrast, CMA eliminates the need for heating, reducing energy use by 71% (80 MJ/t) and emissions to just 4.0 kg CO_2_e/t. These values account for additional energy requirements during the laying process (~16 MJ/t), as shown in Figure 15. As a result, CMA and WMA produce 16–71% lower emissions than HMA [146].

**Figure 16:**
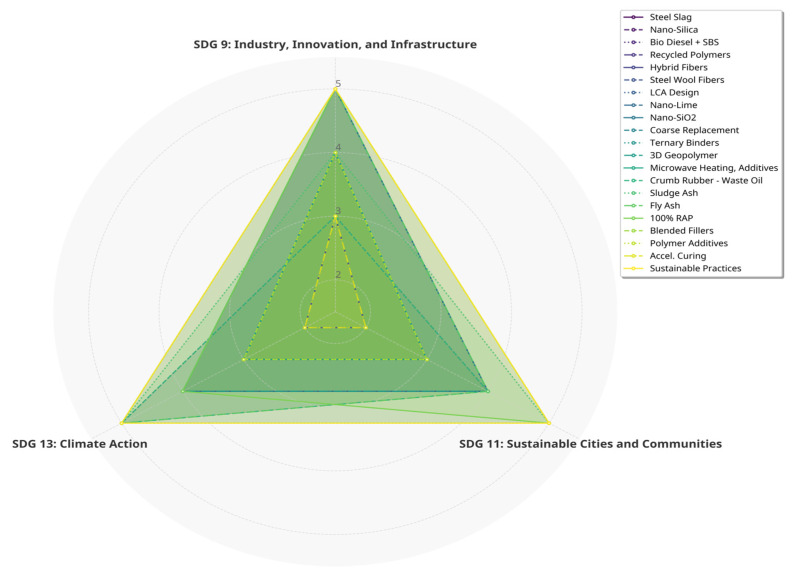
Recent advancements in CMA linked with SDGs 9, 11, and 13.

This combustion releases 22 ± 4 kg of CO_2_ per ton of HMA [N = 20 studies] [145]. In Taiwan, the annual production of 1.5 million tons of HMA results in approximately 39,480 tons of CO_2_ emissions. In addition to CO_2_, asphalt plants emit NO_x_, SO_x_, VOCs, and particulate matter (PM) due to incomplete fuel combustion and bitumen volatilization, negatively affecting air quality and public health. Bitumen refining produces approximately 0.5 tons of CO_2_ per ton of binder; similarly, aggregate production is very energy intensive [147,148]. According to a report by Lokesh from the Decarbon8 initiative [149], the majority of emissions in road construction are generated by materials (i.e., 70 ± 5%), with cement being the largest contributor at 131 kg CO_2_ e/e/ton and asphalt binder second at 40 kg CO_2_ e/e/ton. Transportation generates 10 ± 3% of total emissions, on-site generation of 2–4%, and lighting during operation of 14 ± 5%, while maintenance also contributes an additional 4–6%. Globally, total annual emissions from road infrastructure are estimated to be between 2.5–3.0 billion tons, with each kilometer of road producing between 500–2000 tons of CO_2_ over its lifespan [149]. These results underscore the urgent need to meet global climate change targets. CMA provides a low-emission solution to the problems. Compared to HMA and WMA, CMA shows an average reduction in total GHG emissions of 42.7 ± 3.2%, as exhibited in Figure 15 [146]. CMA provides a technology with no cure that drastically reduces emissions and energy consumption [24,81,146]. This is attributed to its ambient-temperature mixing, elimination of fuel combustion, and the ability to incorporate 100% RAP. For example, Taiwan’s CMA trials demonstrated robust performance, with a 7.7 mm rut depth after 20,000 wheel passes, matching HMA’s durability while avoiding 4,050,000 kWh of annual electricity use for the same production volume [148]. Moreover, CMA avoids the production of toxic by-products such as dioxins and heavy metals. By reusing waste materials such as steel slag and wastewater sludge ash (WSA), CMA supports the circular economy and contributes to environmental targets, including the United Nations Sustainable Development Goals (SDGs), specifically SDG 9, SDG 11, and SDG 13 [150], as shown in Figure 16. Thus, reusing waste materials aligns with the UN Sustainable Development Goals.

1. SDG 11: CMA reduces reliance on virgin resources by incorporating RAP, WSA, and steel slag, thereby reducing landfill disposal and extending pavement life. For instance, using 100% RAP improves fatigue life by 49% and reduces permanent deformation. Steel slag provides a self-healing mechanism, enabling strength recovery of 74%, thus reducing future maintenance [150,151].
2. SDG 9: Advanced materials stabilized by nano-silica, ternary PRBs, and 3D-printable geopolymers promote the mechanical properties of CMA as a basis for durable low-carbon infrastructure solutions.
3. SDG 13: Innovations such as SBS binders with biodiesel blends, fly ashes, RHA, and microwave curing techniques reduce emissions of GHG in the process and enhance the curing and stiffness rates. That is, CKD and soda straw ash enhance the initial strength and reduce the need for heat techniques [148,150].

By focusing on waste reuse, emissions reduction, and industrial innovation, CMA technologies provide a concrete way forward to decarbonizing road construction [148,150].

### 8.1. Innovations in CMA Formulation

Recent advancements in CMAs have focused on their sustainability, mechanical performance, and cost through the inclusion of waste materials, novel additives, and advanced manufacturing methods. These advancements are aimed at the reduction in virgin resource consumption, minimizing environmental impact, and overcoming old deficiencies such as low early strength and moisture vulnerability. The principal advancements are the use of industrial by-products (e.g., sewage sludge ash and steel slag), the use of recycled aggregates, and the implementation of energy-efficient methods of curing.

**Figure 17:**
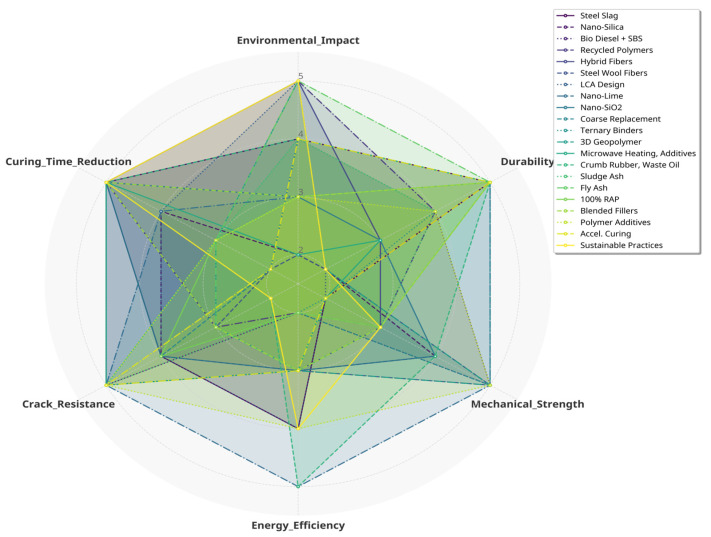
Comparison of key innovations in CMA across sustainability and engineering metrics.

Figure 17 illustrates the novel advancements in the area, taking account of the relative advancements in levels of efficiency relative to important sustainability measures (e.g., reduction in carbon footprint and waste valorization) and in engineering terms (e.g., durability and crack resistance), thus providing a valid overall assessment of their relative value in the area of the modern tabulation of information on the state of the art of pavement technology as applied to CMA. Below are the key advancements:

#### 8.1.1. Waste-Derived Fillers and Aggregates

Table 6 presents the utilization of innovative waste-derived materials employed as fillers and aggregates in CMA, highlighting their potential to enhance performance and promote sustainability in pavement engineering.

#### 8.1.2. Advanced Additives and Binders

Crumb rubber + cement (14% CR + 2% OPC) raised fracture toughness and lowered moisture damage [154]. Cement + fly ash (2% OPC + 1% FA) improved particle bonding and long-term strength in RAP-rich CMA [44].

#### 8.1.3. Low-Energy and Accelerated Curing Techniques

Microwave heating improved workability and shortened curing of cold bitumen emulsion mixtures (CBEMs) [126]. The elevated curing temperatures (40–60 °C) reduced curing time by 45 ± 5% to 7 days, while improving early strength and fatigue resistance [120].

#### 8.1.4. Self-Healing and Durability Enhancements

Microwave-activated steel slag restored up to 70 ± 4% of semi-circular-bend strength; dry/wet/freeze–thaw stiffness gains of 23%, 46%, and 70%, respectively [155]. Cement kiln dust (CKD 1% + OPC 2%) improved moisture resistance, rutting, and fatigue life [156].

#### 8.1.5. Environmental Optimization

Life-cycle assessments (LCAs) reported 20 ± 5% CO_2_ and 86 ± 4% energy savings when recycled materials are used in CMA [156]. Silane-modified granite (KH-560) improved aggregate–binder adhesion to the level of alkaline aggregates [157]. Rice husk ash (RHA 2.5% + GGBFS 1.5%) accelerated curing and early strength, offsetting the low initial stiffness typical of CMA [158].

### 8.2. Sustainability Evaluation Through LCA of CMA Versus HMA

LCAs have comprehensively evaluated the environmental and economic performance of asphalt pavement technologies, including HMA, WMA, CMA, and FMA. Among these, HMA, typically produced at temperatures above 180 °C, is the most energy-intensive. It requires 40.68 ± 2 TJ/km and emits approximately 52 ± 2 kg CO_2_ eq/ton, due to the fossil fuel-based heating of aggregates and binder [146,151,159], as shown in (Figure 1 and Figure 15). WMA, with production temperatures reduced to 100–140 °C, achieved a 35% reduction in energy consumption (34.62 ± 5 TJ/km) and an 11.9 ± 2% reduction in CO_2_ emissions (52 ± 8.2 kg/ton) compared to HMA [146,151]. However, it still requires heating and produces a moderate environmental impact.

By contrast, CMA eliminates the need for heating, resulting in a 64% reduction in energy demand (0.0348 TJ/km) and a 53% reduction in emissions (46,779 kg CO_2_ eq/km) compared to HM. However, its mechanical weaknesses, such as low strength and high moisture susceptibility, limited its application in high-traffic scenarios without additives like cement or FA [10,44,59,146,160]. FMA, which is a type of cold mix asphalt that uses foamed bitumen in place of cutback and/or emulsified asphalt, shows similar environmental advantages. It also shows 40–50% savings in cost compared to traditional rehabilitation methods, with project costs ranging from USD 7940 to USD 9527 per km for this type of cold mix, compared to USD 15,879 per km for HMA. However, it is also subject to delays in curing, as well as sensitivity to moisture [44]. Again, CMA and FMA are generally more economical than HMA. Reported construction costs for CMA were USD 12,193 per km. In contrast, the lowest cost for FMA was attributable to reduced material use and maintenance over the long-term life-cycle cost analysis period [44,59]. Technological advances have overcome past limitations associated with CMA. Use of various additives, such as cement, lime, and/or polymers, has resulted in improvement of the mechanical properties of the cold mix asphalt, which now may be had with Marshall stability values equivalent to those of HMA, after it has been cured, and this shows a reduction in moisture susceptibility of as much as 40 ± 10% [26,45,111].

A recent research study [161] showed that CMA had 8.06% fewer present life-cycle deterministic carbon emissions (5.72 million kg CO_2_-eq/km) than HMA (6.22 million kg CO_2_-eq/km) over a 15-year time frame. Monte Carlo simulations (10,000 experiments) revealed three distinct, non-overlapping distributions for Global Warming Potential (GWP) and energy consumption (EC), indicating a 99.74% probability that CMA has lower GWP and a 99.82% probability that CMA has lower EC than HMA. The key contributors to CMA’s emissions include cement manufacturing (19–24% of GWP), diesel fuel (38–53% of all fuel-related impacts), cement-stabilized base layers (36–37% of total emissions), and recycling procedures (10–18%).

CMA outperformed HMA in terms of sustainability: CMA was demonstrated to have both 72% more energy efficiency than HMA (134 MJ/ton vs. 476 MJ/ton) and 80% fewer emissions (7.1 kg CO_2_/ton vs. 35.5 kg CO_2_/ton) [162]. In addition, CMA saved 64% in energy (34,806 MJ/km vs. 95,845 MJ/km) and 53% in CO_2_ emissions and utilized 23% less materials when used on rural roads [94]. CMA was shown to be cost-effective as well; CMA had 57% less in material costs (USD 150.00/ton vs. USD 346.00/ton) and 23% less in net present worth than HMA [161,162,163,164]. Because of these environmental, economic, and technical benefits, CMA is being promoted by the US, EU, and Indian Governments as a “green” pavement option using procurement options and carbon credits [44,59].

## 9. Performance Limitations and Field Implementation Challenges of CMA

Despite advances in modern CMA technologies, several inherent limitations hinder widespread adoption. These challenges, if not properly managed, can lead to suboptimal field performance and reinforce historical skepticism. Key drawbacks include curing behavior, moisture sensitivity, long-term durability, and field variability [139,140,142,143,144,146].

### 9.1. Slow and Climate-Dependent Curing

This is a characteristic of CMA because its curing process is dependent upon the rate at which water evaporates from the surface of the material; consequently, it is very temperature- and humidity-sensitive [120]. The time that CMA takes to develop strength is generally longer than that of HMA. CMA may take days to months to cure, depending upon the environmental conditions (i.e., <10 °C). While additives such as cement may help to improve early strength development, they will not eliminate the material’s dependency upon environmental factors [33,120,154,158].

### 9.2. Moisture Sensitivity and Adhesion

The early CMA blends generally had poor resistance to moisture (low TSRs < 70%), primarily as a result of weak binding properties between aggregate particles when exposed to water [2]. Although today’s blends typically have TSRs greater than 85%, they also depend upon correct blend designs and anti-stripping agents or active filler additions; however, residual moisture can inhibit adhesion if the emulsion does not completely break down [154].

### 9.3. Long-Term Durability Uncertainties

Although some CMA sections have performed well for more than 15 years, data for high-traffic conditions remain limited [140]. CMA binders undergo different aging mechanisms than HMA and may be susceptible to alternative forms of degradation. Additionally, inconsistencies between laboratory aging protocols and real-world conditions make long-term performance predictions uncertain [139,140].

### 9.4. Field Variability and Contractor Experience

CMA performance is highly sensitive to production and construction practices.

1. Moisture-induced stiffness scatter: Chongzheng Zhu [104] quantified the impact of stockpile moisture on 120 plant-produced CMA batches containing 35% RAP. A 1% increase in RAP free water elevated the effective binder content by 0.14% and reduced in situ air voids by 1.8%. Tensile adhesion decreased by 28% when the overnight relative humidity exceeded 85%. To mitigate this variability, contractors now enforce a 0–2% moisture limit and employ microwave sensors to adjust flux-oil dosage in real time [104].
2. Temperature-driven viscosity window: A study reported by Ding et al. [7] recorded binder viscosity at 5 min intervals during 42 roadside trials under ambient temperatures ranging from 5 to 35 °C. Viscosity at 60 °C ranged from 1.1–2.0 Pa·s (CV = 18%), and the 1.6 Pa·s pot-life threshold was exceeded in 26% of loads, resulting in an average increase of 0.6 mm in Hamburg rutting depth. Consequently, a weather specification (substrate temperature ≥ 5 °C, relative humidity ≤ 85%) has been adopted to control field variability [7].
3. Compaction variability at low temperature: Low temperature can result in a stiffer material (CMA), which makes the material less compactable; therefore, it has an uneven density and greater air voids. The results of these characteristics will lead to lower strength and less durability. It is necessary to adjust the appropriate mix design and optimize the compaction strategy to limit the variation that occurs with the temperature [83,133].

The lack of standardization in the construction process and the experience of contractors make this problem worse for many [96,98,101,102,112,113,115,116,117,118,119].

## 10. Conclusions and Future Direction

CMA represents a radical shift in pavement engineering, combining environmental advantages with economic advantages. By eliminating the need for high-temperature production processes, CMA achieves up to 64% energy savings and increases CO_2_ emissions by over 50% compared to HMA. This enables compliance with global decarbonization and net-zero requirements. Another enhancement to CMA is the incorporation of RAP materials, bio-based rejuvenators, and nano-additives, which improve CMA’s mechanical properties, producing strength, stability, and durability comparable to HMA and thereby aligning with the principles of the circular economy. Notwithstanding progress in CMA, many problems remain, particularly those relating to strength gain at early ages, moisture susceptibility, and inconsistent field-cure behavior. However, a significant barrier to success exists in that there is no approved globally standardized mix design protocol, with consensus lacking regarding current practices (e.g., AI MS-14) and Technical Guidelines (TGs), AASHTO PP 80-20, IRC: SP:100 [97,98], differing greatly with respect to gradation control, coating requirements, curing regimes, and evaluations. Greater standardization can be ensured by establishing a globally harmonized, performance-based mix design protocol incorporating (1) the traffic-based classification hierarchy of TG; (2) the RAP incorporation and coating requirements specified by AASHTO PP 80-20; (3) the empirical gradation and compaction relationships available in AI MS-14; and (4) the curing and stability evaluations specified by IRC: SP:100, from a field outcome perspective. Combining all these factors into a unified standardized practice incorporating laboratory reproducibility, field strengths, and climatic adaptability should be sufficient to achieve success. The utilization of nano-engineered binders, cementitious fillers, microwave-assisted curing, and AI-predictive modeling will continue to advance the sustainability of CMA performance. A formalized research approach of five years is proposed to address problems of this nature:

- Phase 1 (2025): Optimization of nano-additives (graphene oxide and nano-silica) to improve adhesion and moisture resistance.
- Phase 2 (2026–2027): AI-based forms predictive of the curing kinetics, the RAP–binder interaction, and the climate-dependent performance.
- Phase 3 (2028–2030): Utilization of IoT-enabled systems employing smart pavements and self-healing storms.

At the same time, supportive policy frameworks, such as the European Union’s green purchasing schemes and the emerging carbon credit initiatives in India, China, and the USA, are expected to align with the increasing integration of universal CMA. Regional development worldwide, exemplified by Sweden’s cement-free emulsion (4–4.8% bitumen), provides tangible evidence of reforms that can be adopted in regulatory and climate change practices to promote international trade. The final obstacle is the considerable gap between laboratory research and field application. Establishing unified standards and performance-based testing methods will be vital for progressing CMA from limited repair uses to broader infrastructure systems. Through international collaboration, technological advancement, and the standardization of practices, CMA is poised to define the next generation of low-carbon, intelligent, and resilient pavement infrastructure. In doing so, CMA will make significant contributions to the UN Sustainable Development Goals: 9 (Industry, Innovation and Infrastructure Development), 11 (Sustainable Urban and Community Development), and 13 (Climate Action).

## Figures and Tables

**Table 2:** Comparison of bituminous mix design standards.

Category	AI MS-14 [65,72]	TG [96]	AASHTO PP 80-20 [99]	IRC: SP:100 [97,98]
Blend Classification	Well-graded or gap-graded aggregates	Traffic-dependent categories: BSM1 (>6 MESA), BSM2 (<6 MESA), BSM3 (<1 MESA).	Requires 100% crushing RAP to meet gradation targets; permits ≤15% virgin aggregates to correct deficiencies.	BM and SDBC mixes are specified.
IRAC/IEC Calculation	Empirical formulas with critical sieve sizes at 2.36 mm and 0.075 mm.	Not specified	Not specified	An empirical approach using 2.36 mm and 0.090 mm sieves as breakpoints.
Coating Requirements	≥50% aggregate coating mandated.	Not specified	An aggregate coating of ≥90% is mandated, assessed visually during mixing.	Visual inspection for adequate coating (no quantitative threshold).
OTLC Determination	Derived from the moisture content yielding maximum dry density.	Optimum moisture content is established using modified AASHTO compaction.	Optimum water content determined at maximum dry density (modified Proctor). OTLC = Optimum water + foamed asphalt content.	Not specified
Variation in RAC	Maintains a constant OTLC	Not specified	Test ≥3 emulsion contents (e.g., 3.0%, 3.5%, 4.0%); select optimum via stability/voids.	Maintains the same OPWC, leading to a gradual increase in TLC.
Curing Process	Dry stability: 24 h mold (25 °C) → 24 h oven (40 °C) → 24 h mold (25 °C). Soaked: 48 h water immersion	Level 1: 72 h at 40 °C (unsealed). Levels 2–3: 26 h at 30 °C → sealed → 48 h at 40 °C	72 h at 40 °C → 24 h at 25 °C (simulates 14-day field curing).	Air-dry loose mix (1–2 h) → oven-dry (40 °C, 2 h) → compact → 24 h mold (25 °C) → 72 h oven (40 °C).
Determination of ORAC	Maximizes soaked stability and dry density while meeting other criteria	Levels 1–2: indirect tensile strength (ITS) tests. Level 3: triaxial test results	Not Specified	It mainly focuses on maximum dry stability and density; soaked stability is not considered.
Moisture Damage	Stability values that have been retained are evaluated.	TSR values alongside moisture sensitivity tests are conducted	Stability values that have been retained are evaluated.	Analysis of retained ITS values.

**Table 3:** Studies related to the performance of CMA.

Reference	Type of Emulsion/Asphalt	Blend Overview	Dosage	Curing	Key Results	Summary
Chongzheng Zhu [104]	CSS	RAP 84% + 12% virgin agg. + 4% mineral powder + 1.5% cement, FA, RH	1.5% cement, FA, RH	2 days @ 60 °C	ITS: 0.75–1.04 MPa; Stability: 1800–4200 passes/mm; TSR: 75–85%	FA maximized CRM tensile and low-temp performance; 0.75% FA + cement cut CO_2_ by ~50%.
Wenting Yang [33]	CSS	RAP 70% + limestone 30%; 4.4% added water	4% emulsion; 0–2% cement	2 days @ 60 °C, 3 days @ 20 °C + 2 days @ 60 °C	ITS: 0.74–0.94 MPa; AV: 10.3–11.7%; CSED: 2.03–2.77 kJ/m^3^	Staged curing prioritizes cement hydration, producing a denser, stiffer matrix.
Li Yawen [49]	CSS	Aggregates + filler + cement (0–6%)	8% emulsion + 0–6% cement	28 days @ 23 °C, 55% RH	ITS: 540–1250 kPa; Stability: 6.8–13.1 kN; AV: 9–10%	2% cement provides ≥80% of 28-day strength in 7 days; higher cement accelerates early strength.
Nassar et al. [105]	CE (C60B5)	Cold asphalt + OPC, FA, GGBS, Silica Fume	OPC: 8.8–43.9 g; additives 20–40%	-	ITSR: 80–105%; Stiffness: 282 MPa; AV: 8.5–9.8%	GGBS + SF reduces porosity and improves stiffness and durability.
Dulaimi [114]	CSS (C50B4)	6% total filler: 4% GGBS + 2% CCR	4% GGBS + 2% CCR	3, 7, 56 days @ 20 °C	ITS: 1540–2510 MPa; AV: 8.9–9.2%; Rut: 3.2–3.5 mm; ITSR: 86–88%	4% GGBS + 2% CCR outperforms limestone mixes, matches hot mix stiffness in 3 days.
Zhu Siyue [106]	SBS- Modified Emulsified Asphalt	CEAM + Cement + Fibers	3% OPC, 0–0.2% fiber	3–7 days @ 20 °C	ITS: 0.42–0.91 MPa; Flexural: 0.60–1.18 MPa; ITSM: 1050–2240 MPa; Rut: 2.5–4.8 mm; ITSR: 72–90%	0.2% fiber + 3% OPC increases ITS, fatigue life, and reduces rut depth.
Chegeniza-deh [81]	CSS	100% RAP + BE	2–4% BE	1–12 weeks @ 20 °C, soaked 24 h @ 25 °C	ITS: 230–561 kPa; RM: 771–2510 MPa; Rut: 9–13.6 mm; Fatigue: 102–153 k cycles	4% CSS + 100% RAP optimizes stiffness, strength, fatigue, and rutting.
Rezaei [2]	Cutback/Emulsified/ Polymer-Modified	9 cold mixes: DG + OG	Binder 2.4–6.7%	24 h @ 25 °C, oven-cured 18 h @ 135 °C	MS: DG: 6.8–19 kN; OG: 3.4–10.1 kN; ITS: 370–1568 kPa; TSR: 0.64–1.06	DG cold mixes with a low dust-to-binder ratio yield higher stability and the lowest rutting/moisture damage.

CSS: cationic slow-setting emulsion; RH: rice husk ash; OPC: Ordinary Portland Cement; GGBS: Ground-Granulated Blast-furnace Slag; CSED: Crack-Strain Energy Density; RM: resilient modulus; AVs: air voids; @: at.

**Table 4:** Three-stage strength development (cement-modified CMA) [49].

Mix ID	Cement (%)	Stage	Time (d)	Marshall Stability (kN)	ITS (kPa)	ITSM (MPa)
C0	0	I	0–3	2.0	250	520 (36%)
		II	3–14	4.8	600	1360 (95%)
		III	14–28	5.7	676	1435
C2	2	I	0–3	5.0	370	3500 (52%)
		II	3–14	9.2	720	6300 (93%)
		III	14–28	10.5	807	6764
C4	4	I	0–3	7.5	680	6300 (46%)
		II	3–14	15.2	1220	13,400 (97%)
		III	14–28	16.0	1331	13,805
C6	6	I	0–3	13.3	730	8200 (46%)
		II	3–14	18.7	1420	16,100 (91%)
		III	14–28	21.0	1577	17,657
HMA	—	—	—	—	—	2705

Stage I = rapid emulsion break + early cement hydration; Stage II = slower strength gain from continued curing; Stage III = property plateau; numbers in parentheses (%) represent proportion of the 28-day value (Stage III); HMA value shown only for ITSM comparison at 28 days.

**Table 6:** Performance and Sustainability Benefits of Waste-Derived Fillers and Aggregates in CMA.

Material	Key Finding	Performance Gain	Sustainability Gain	Source
Wastewater sludge ash (WSA)	Replaces limestone filler; passes UK/EN leachability limits	Increase moisture resistance and durability	Eliminates calcination CO_2_	[152]
RAP	50% RAP > control stability; 100% RAP increased +49% fatigue life; decrease rut depth	Matches or exceeds virgin mix	Diverts waste; cuts virgin aggregate	[44,81]
Hybrid (50% RAP + 30% other recycled agg.)	Portland cement/bitumen emulsion binder achieves parity with HMA	Stable, durable mix	Reduces virgin content by ≥80%	[27,83,153]

## Data Availability

No new data were created or analyzed in this study. Data sharing is not applicable to this article.

## Nomenclature

CMA	Cold Mix Asphalt
RAP	Reclaimed Asphalt Pavement
MS	Marshall Stability
IRAC	Initial Residual Asphalt Content
CSS	Cationic Slow-Setting (emulsion)
SF	Silica Fume
HMA	Hot Mix Asphalt
TSR	Tensile Strength Ratio
MQ	Marshall Quotient
IEC	Initial Emulsion Content
OPC	Ordinary Portland Cement
FA	Fly Ash
WMA	Warm Mix Asphalt
ITS	Indirect Tensile Strength
LCA	Life-Cycle Assessment
OTLC	Optimum Total Liquid Content
GGBS	Ground-Granulated Blast-furnace Slag
CKD	Cement Kiln Dust
